# Electrocatalysis by Coinage Metal Nanoclusters of Atomic Precision: Tailoring Catalytic Reactivity and Stability by Ligands and Composition

**DOI:** 10.1002/asia.202500985

**Published:** 2026-01-14

**Authors:** Yingwei Li, Ekin Ozel, Rachel A. Jun, Rongchao Jin

**Affiliations:** ^1^ Department of Chemistry University of Hawaiʻi at Manoa Honolulu Hawaiʻi USA; ^2^ Department of Chemistry Carnegie Mellon University Pittsburgh Pennsylvania USA

**Keywords:** atomically precise nanoclusters, coinage metals, CO^2^ reduction, electrocatalysis

## Abstract

Atomically precise coinage metal nanoclusters (NCs) have emerged as a powerful platform for uncovering structure–property relationships and for various applications. aOwing to their well‐defined atomic structures, discrete electronic states, and tunable surface environments, these NCs enable systematic studies of active‐site modulation at the atomic level, which is especially important for nanocatalysts and has long been pursued in heterogeneous catalysis. This review provides a comprehensive overview of recent advances in the electrocatalytic applications of coinage metal NCs protected by thiolate, phosphine, amine, and alkynyl ligands. In addition to the size dependence, key effects—ligand types, morphology, core doping, and surface modification—on the CO_2_ reduction reaction (CO_2_RR) are discussed first. Then, cases of non–alkynyl‐protected NCs with exceptional CO_2_RR activities are illustrated to show how atomic packing, ligand engineering, lattice hydride, and alloying can be used to design high‐performance NC catalysts. Cu‐based NCs are highlighted since value‐added multicarbon products can be created in CO_2_RR. The review then discusses how alkynyl protection introduces unique metal–ligand interfacial structures via σ–π anchoring, leading to reduced ligand coverage and increased exposure of active sites of metal. Recent progress in alkynyl‐protected NCs has expanded the accessible structural library, enabling efficient electrocatalysis for CO_2_RR, nitrate reduction (NO_3_^−^RR), and hydrogen evolution reaction (HER). The synergistic effects of bimetallic compositions, ligand functionalization, and nanocluster architectures are examined in detail, illustrating how subtle changes in surface chemistry translate into dramatic improvements in catalytic performance. Through comparisons between non–alkynyl‐ and alkynyl‐protected NCs, this review underscores the central role of surface chemistry in tailoring electrocatalytic activity, selectivity, and stability. Finally, future directions are outlined, emphasizing the importance of combining atomic‐level structural precision with rational ligand engineering and heteroatom doping to design next‐generation electrocatalysts.

## Graphical abstract

Atomically precise coinage metal nanoclusters offer an exceptional platform for probing structure–activity relationships in electrocatalysis. This review first outlines general catalyst‐design principles, emphasizing how ligand environment, morphology, doping, and surface modification govern electrocatalytic behavior. We then compare non‐alkynyl and alkynyl systems, highlighting how ligand anchoring and engineering enhance active‐site exposure. Special attention is given to Cu‐based nanoclusters, which uniquely enable value‐added C_2_ product formation, illustrating their potential for advanced CO_2_ conversion.

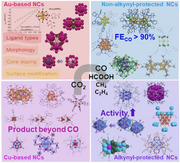

## 1 Introduction

Electroreduction offers a controllable and scalable technique for converting protons and small molecules—such as N_2_ and carbon dioxide—into valuable fuels and chemicals under mild conditions [1, 2]. Compared to intermittent renewable electricity, electrocatalysis serves not only as a method of chemical synthesis but also as a means of storing intermittent energy in stable chemical bonds [3, 4]. Among various electroreduction targets, CO_2_ has attracted significant attention due to its nature as a primary greenhouse gas of concern and its abundance as an industrial waste product. Thus, electrochemical CO_2_ reduction reaction (CO_2_RR) presents a promising approach to mitigating atmospheric CO_2_ levels and simultaneously storing renewable energy in the form of energy‐dense chemical products. This process enables the conversion of CO_2_ into fuels and feedstocks such as carbon monoxide, formic acid, hydrocarbons, and alcohols—compounds of high industrial value [5, 6, 7]. The appeal of CO_2_RR lies in its potential sustainability: it operates at ambient temperature and pressure and can utilize non‐noble metal catalysts, and is also compatible with aqueous electrolytes. However, several technical challenges still remain, including the inherent difficulty in CO_2_ activation due to the high stability of C─O bonds (806 kJ mol^−1^), the need for high overpotentials [8], poor product selectivity, and undesirable competition with the hydrogen evolution reaction (HER), which severely limits the efficiency and practical implementation of the CO_2_RR process [9, 10]. To overcome these limitations, it is important to rationally design efficient and selective electrocatalysts capable of promoting CO_2_ adsorption, stabilizing reaction intermediates, and facilitating product desorption without excessive energy input. Continued advances in catalyst engineering and mechanistic understanding are essential to realizing the practical potential of electrochemical CO_2_ conversion as a key component of the grand goal of a carbon‐neutral energy future.

Atomically precise coinage metal nanoclusters (NCs) are compositionally well‐defined and structurally precise materials with a core diameter < 2 nm and unique molecule‐like properties and discrete electronic energy levels, which are distinctive from the much larger counterpart—metal nanoparticles with plasmon resonances. The NCs materials, since their first synthesis, have been widely applied to various catalytic processes [11, 12] because they not only possess free valence electrons (though with quantized energy)—endowing them with attractive catalytic properties—but also feature specific sizes and well‐defined structures (as opposed to polydisperse or relatively monodisperse nanoparticles) and defect‐free surfaces (unlike regular nanoparticles with imperfections), as revealed by single crystal x‐ray diffraction [13, 14].

The development of many series of atomically precise Au NCs has provided opportunities to study the size effect on various catalytic reactions [15, 16]. The availability of precise structures makes it possible to divide target NCs into two series according to the thiolate ligand used (resulted in different kernel structures): 2‐phenylethanethiolate (PET)–protected NCs (icosahedral Au_38_, Au_144_ and Au_333_) and 4‐tertbutyl‐benzenethiolate (TBBT)–protected NCs (Au_28_, Au_36_, Au_279_)—to study the size effect on CO_2_RR systematically. The smaller‐sized members of both series showed higher *j*_CO_ and ∼100% FE_CO_ at potentials > −0.8 V due to a higher S:Au ratio or active sites, while larger metallic NCs (i.e., Au_333_ and Au_279_) gave lower FE_CO_ [17]. It seems that size‐dependency is inherited at ultrasmall sizes when a metallic to nonmetallic (molecular‐like) transition happens. However, the relationship becomes more complex upon closer examination [15].

Gold‐based catalysts are known for their high selectivity toward CO in CO_2_RR. However, conventional gold nanostructures, even highly monodispersed, are not identical when examined at the atomic level, making it difficult to establish a clear structure–activity relationship. The atomically precise coinage NCs started from thiolate‐protected Au NCs [18, 19, 20], and they were thus applied to electrocatalysis in early research to address the size dispersity issue [11, 21]. Herein, we highlight several state‐of‐the‐art examples that demonstrate remarkable CO_2_ conversion performance.

In contrast to conventional nanoparticles—for which a clear nanometer‐level size‐dependent activity/selectivity trend has been demonstrated in electrocatalysis [27, 28, 29], it is still difficult to draw conclusions on the size‐dependency for small clusters (gas phase made) with very narrow differences in atomicity, and atomicity dependency was observed early on in cluster electrocatalysis. Among Pt*_n_* clusters without ligands formed by physical deposition, Pt_4_ and Pt_10_ clusters were the most active for ethanol oxidation [30], while Pt_7_, Pt_10,_ and Pt_11_ clusters rivaled conventional nanoparticles in oxygen reduction reaction (ORR) [31]. Dendrimer‐templated Pt clusters confirmed this size sensitivity, with Pt_12_ outperforming both larger clusters and nanoparticles, and even doubling the activity of Pt_13_ due to a single‐atom‐induced geometric rearrangement [32, 33, 34].

**SCHEME 1:**
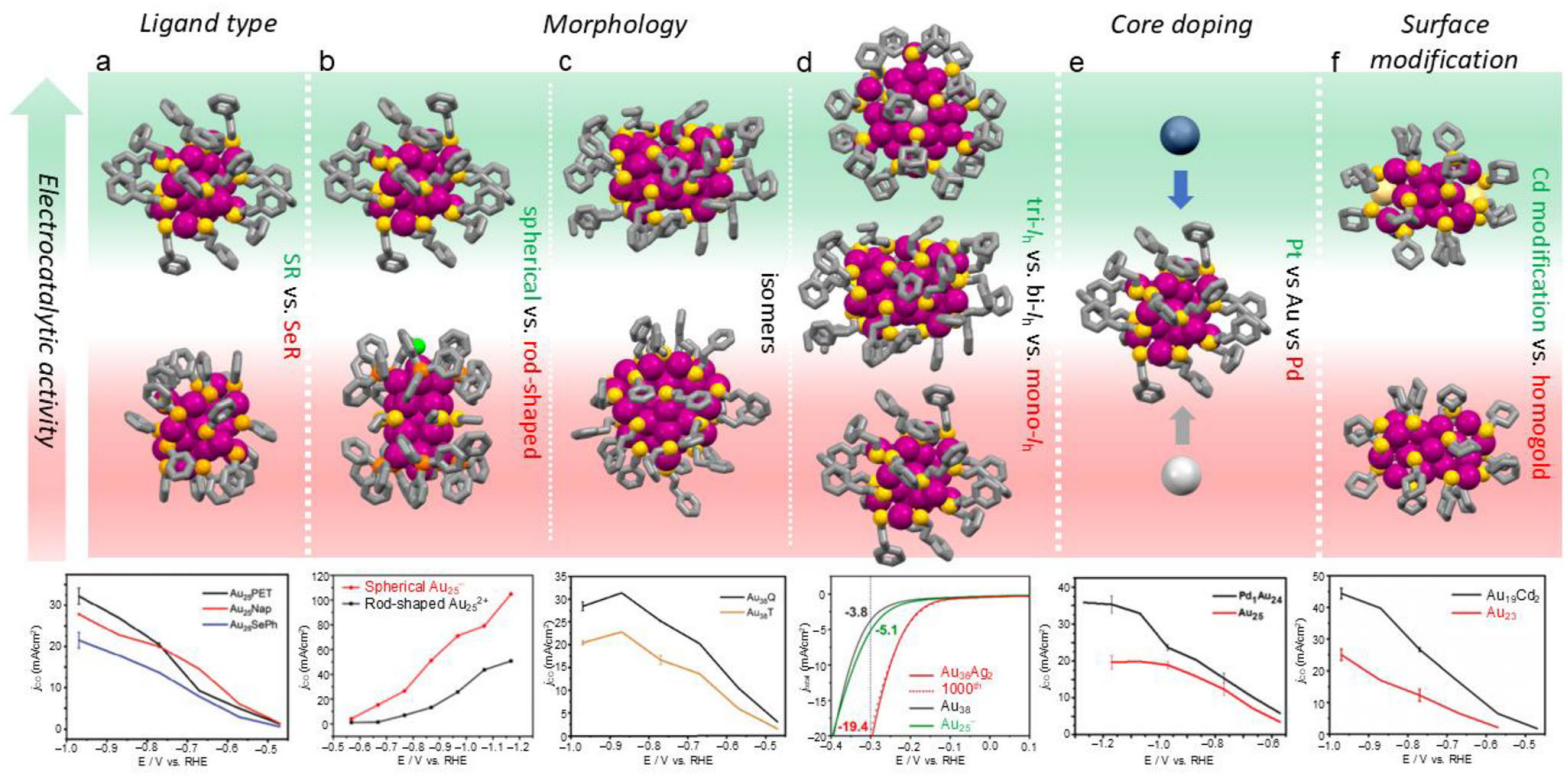
Factors that affect the electrocatalytic properties of Au‐based NCs with small size differences. (a) Ligand effect of SR and SeR on Au_25_ [22]. Copyright 2021, The Royal Society of Chemistry. Morphology effect of (b) spherical vs. rod‐shaped Au_25_ [23], Copyright 2018, American Chemical Society; (c) two isomeric Au_38_ [17], Copyright 2022, Wiley‐VCH GmbH; (d) mono‐*I*_h_, bi‐*I*_h,_ and tri‐*I*_h_ NCs [24], Copyright 2021, American Chemical Society. (e) Core doping effect on Au_25_ [25]. Copyright, 2020 American Chemical Society. (f) Surface modification effect on Au_23_ [26]. Copyright 2020, Wiley‐VCH GmbH.

However, these clusters still show a relatively narrow range of sizes in mass spectrometry, which is not as precise as the newly developed ligand‐protected nanoclusters synthesized by atomically precise nanochemistry [35]. With full structural information—revealing everything from the atomic packing of the metal core to the metal–ligand interface structure and surface ligand arrangement [35], more factors in addition to size or atomicity, have been found to be critical in determining the catalytic activity. Here are some important ones for understanding the structure–property relationships:

(1) **Ligand types**: The hindrance at the Au NC catalyst–support interface plays a critical role in some reactions, such as the CO oxidation [36], and the local microenvironment is also true when applying atomically precise NCs in CO_2_RR. When comparing thiolate‐protected Au_25_ with selenite‐protected counterpart, the Faraday efficiency for CO (FE_CO_) of the former was found to be almost 100% but the latter only showed FE_CO_ of ∼80% both at −0.78 V vs. RHE, whereas the type of thiolate did not show obvious impact on the activity (Scheme 1a) [22].

**FIGURE 1:**
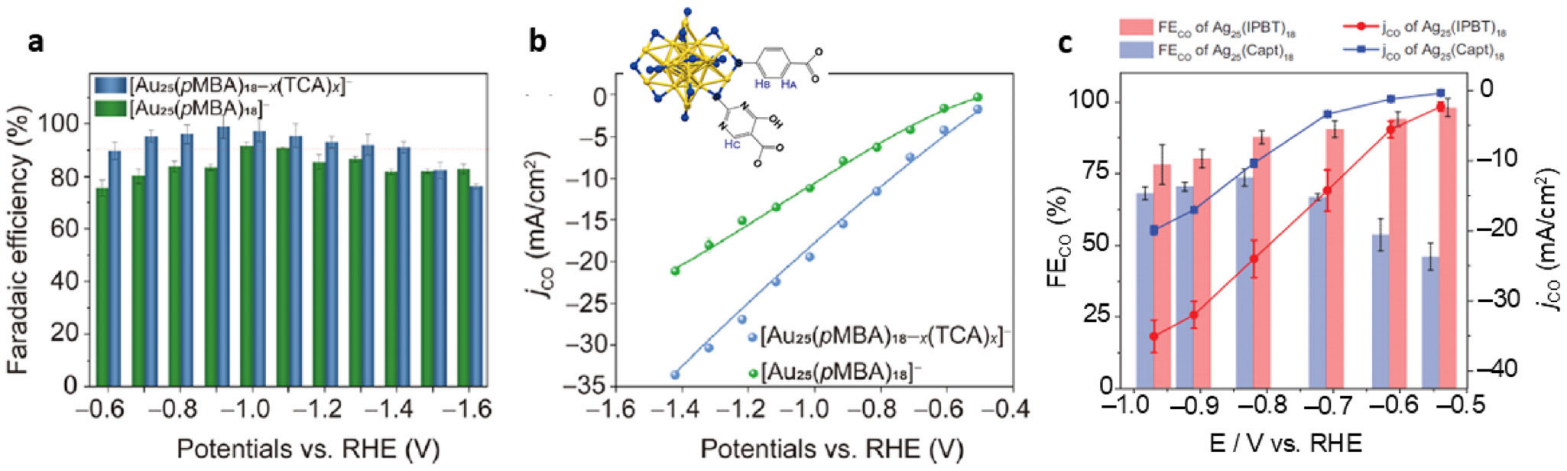
(a) Faradaic efficiency for CO and (b) CO partial current density at different potentials catalyzed by [Au_25_(*p‐*MBA)_16_(TCA)_2_]^‒^ NCs [37]. Copyright 2024, American Chemical Society. (c) FE_CO_ and *j*_CO_ of Ag_25_(IPBT)_18_ and Ag_25_(Capt)_18_ at different potentials [38]. Copyright 2023, Wiley‐VCH GmbH.

Inspired by enzyme catalysis, ligand engineering strategies analogous to enzyme catalysis were applied to Au_25_ NCs. Two 2‐thiouracil‐5‐carboxylic acid (TCA) ligands were incorporated into the pocket‐like cavities in the ligand shell of Au_25_, creating [Au_25_(*p‐*MBA)_16_(TCA)_2_]^‒^ (*p‐*MBA = para‐mercaptobenzoic acid) [37]. The TCA ligands, featuring nucleophilic pyrimidine nitrogen, enhanced local CO_2_ concentration near the active Au sites via supramolecular interactions, significantly boosting CO_2_RR performance with a remarkable FE_CO_ of 98.6% and a turnover frequency (TOF) of 39 s^‒1^ at ‒0.9 V (Figure 1a,b). The catalyst also demonstrated long‐term stability over 18 h of continuous operation [37].

The silver analog of Au_25_, that is, Ag_25_ NCs, one protected by hydrophilic captopril thiolate (Ag_25_(Capt)_18_) and the other by hydrophobic 2‐isopropylbenzenethiolate (Ag_25_(IPBT)_18_), were applied to convert CO_2_ to CO [38]. It was observed that the hydrophobic Ag_25_(IPBT)_18_ achieved FE_CO_ over 90% in the potential range from −0.5 V to −0.7 V, outperforming the hydrophilic counterpart (Figure 1c). Moreover, Ag_25_(IPBT)_18_ delivered a remarkable *j_CO_* of 240 mA cm^−2^ at −3.4 V with 91.8% FE_CO_ and maintained stability over 120 h [38]. Operando spectroscopy and DFT revealed that the hydrophobic ligand shell creates a favorable interfacial water structure, leading to weaker *CO intermediate binding, and thus facilitating CO desorption.

(2) **Morphology**: Spherical [Au_25_(SR)_18_]^–^ and rod‐shaped [Au_25_(SR)_5_(PPh_3_)_10_Cl_2_]^2+^ were used to investigate the impact of atomic‐level morphology on CO_2_RR [23]. At all applied voltages, spherical Au_25_ NCs exhibited higher FE and total current density for CO compared to rod‐shaped Au_25_ NCs (Scheme 1b). Specifically, under a potential of −0.87 V, spherical Au_25_ achieved an FE_CO_ of 75% with a current density of 50 mA cm^−2^, whereas its rod‐shaped counterpart showed only 60% and 15 mA cm^−2^ under identical conditions [23]. DFT analysis attributed the enhancement to the sphere's inherent negative charge and the energetically favorable creation of an active site via ligand removal, which stabilizes the key *COOH intermediate better.

When comparing two Au_38_(SR)_24_ isomers, that is, Au_38_Q (icosahedral kernel) vs. Au_38_T (icosahedral + *fcc* kernel), the former maintained approximately 100% FE_CO_ at potentials > −0.87 V and a *j*_CO_ that was 1.5 times higher than the latter (Scheme 1c). DFT calculations indicated that the disparity stemmed from the differences in ligand removal energy, with the average *COOH formation energy on Au_38Q_ (0.17 eV) significantly lower than that on Au_38_T (0.31 eV) [17].

In another case, when comparing [Au_25_(SR)_18_]^–^ with a mono‐icosahedral (*I*_h_) core, Au_38_(SR)_24_ with a bi‐*I*_h_ core and Au_36_Ag_2_(SR)_18_ with a tri‐*I*_h_ core for HER, the current density of Au_36_Ag_2_(SR)_18_ at low overpotential of −0.3 V vs RHE was 3.8 and 5.1 times that of [Au_25_(SR)_18_]^−^ and Au_38_(SR)_24_, respectively (Scheme 1d), due to its low ligand‐to‐metal ratio, low‐coordinated Au atoms and unfilled superatomic orbitals, thus providing a new strategy for constructing highly active catalysts from inert metals [24]. The atomically precise NCs provide far more details on morphological effects that govern catalytic activity in CO_2_RR, in addition to the general size effect.

(3) **Core doping**: Based on the spherical Au_25_ template, replacing the central atom with a single palladium atom was found to drastically enhance the CO_2_RR performance, achieving nearly 100% FE for CO production over a wide potential range from −0.9 to −1.2 V, with a *j*_CO_ of 18.8 mA cm^‒2^ [25], while the FE_CO_ of Au_25_ began to decline beyond −0.9 V due to increased competing HER. Furthermore, PdAu_24_ demonstrated approximately twice the product formation rate of Au_25_ (∼1770 mA/mg vs. ∼980 mA/mg at −1.2 V) and maintained excellent stability during extended electrolysis (Scheme 1e). Theoretical simulations found that Pd doping suppressed thiolate ligand desorption to expose undercoordinated Au sites that favor HER.

By contrast, although electrochemically reducing CO_2_ to syngas using Au_25_ showed high CO selectivity with a FE_CO_ exceeding 95% at low overpotentials (−0.3 to −0.6 V), monoplatinum‐doped PtAu_24_ was found to favor HER [39]. Using this complementary selectivity, simply mixing Au_25_ and PtAu_24_ NCs on an electrode enabled precise and predictable tuning of the H_2_/CO ratio from 1:1 to 4:1 at a constant potential [39], which holds potential in subsequent generation of CH_3_OH via thermocatalysis as electrocatalytic CH_3_OH generation is still challenging.

In the bi‐icosahedral series of Au_38_, Pt_1_Au_37_ and Pt_2_Au_36_ NCs, calculations found that asymmetric central doping with one Pt atom elevated the energy of the highest occupied molecular orbital and was accompanied by the loss of one valence electron, leading to electron‐spin‐induced high activity for CO_2_ electroreduction (FE_CO_ = ∼80%) at −0.6 V, compared to the other two NCs [40].

(4) **Surface modification**: Precise control over the composition and structure of metal NCs is crucial for optimizing their electrocatalytic properties. Cd doping has been found to effectively modify the surface structure so as to boost the CO_2_RR activity toward CO without altering the core structure. Examples are introduced below.

A two‐phase antigalvanic reduction method led to a bimetallic Au_47_Cd_2_(TBBT)_31_ (Au_47_Cd_2_) NC from a Au_44_(TBBT)_28_ (Au_44_) precursor, yielding a NC with two surface Cd atoms integrated into the motifs [41]. In CO_2_RR, Au_47_Cd_2_ exhibited a high FE of 96% for CO production at a low potential of −0.57 V, surpassing the precursor Au_44_ NC and Au nanoparticles (FE_CO_ = 83% and 76%, respectively). This bimetallic NC also showed a superior CO mass activity of −55.6 A g^‒1^ [41]. DFT calculations indicated that the surface Cd atoms stabilize the key *COOH intermediate by forming a Cd–O bond, and thereby lowering the reaction energy barrier and suppressing HER [41].

In another case, an atomic‐level surface modification was performed on a Au_23_(SR)_16_ template [26]. Substituting two surface Au atoms with one Cd atom on both sides of the NC generated Au_19_Cd_2_(SR)_16_ (Scheme 1f), which was further tested in CO_2_RR. Au_19_Cd_2_ exhibited a high FE_CO_ of 90% to 95% and much higher *j*_CO_ within a wide potential range of −0.5 V to −0.9 V, effectively doubling the selectivity of the pristine Au_23_ NC [26]. Furthermore, this bimetallic NC achieved an outstanding mass activity of 2200 mA mg^−1^ at −1.0 V. DFT calculations suggested that the active site is a sulfur atom exposed upon partial ligand removal, and the superior activity was attributed to a significantly lower thermodynamic barrier (0.74 eV) for the formation of *COOH intermediate on Au_19_Cd_2_. This work demonstrates that atomic‐level surface modification is a powerful strategy for boosting catalytic performance.

Following those discoveries, a series of Au‐Cd NCs, including Au_24_Cd, Au_19_Cd_3,_ and Au_38_Cd_4_, were employed to identify the active sites for CO_2_RR [42]. This work demonstrated that the catalytic activity could be precisely controlled by selectively cleaving Au–SR or C–S bonds, a process regulated by cadmium doping [42]. The C–S bond cleavage created open sulfur sites favorable for CO_2_ activation, while Au–SR bond breaking exposed metal sites that promote the competing HER. Among this series, Au_24_Cd exhibited superior performance with FE_CO_ of 90% at −0.5 V, a *j*_CO_ of 18.1 mA cm^−2^ at −0.6 V, and a mass activity of 68.5 A/g_metal_ [42].

## 2 Selected Cases of Non–Alkynyl‐Protected Nanoclusters for Electrocatalysis

In addition to the above‐discussed effects, other coinage metal NCs stabilized with different ligands, including thiolates, alkynyls, phosphines, amines, and carbenes, have also been reported to show high CO_2_ reduction activity with FE_CO_ over 90%. The hydrophobicity or hydrophilicity of ligands can influence the substrate accessibility, intermediate adsorption, and consequently the overall catalytic performance. The atomically precise NCs discussed in this mini review generally possess hydrophobic ligand shells. It is well established that a moderately hydrophobic microenvironment can greatly enhance the diffusion of gaseous species (such as CO_2_), thereby accelerating reaction kinetics [43, 44]. The following cases highlight representative examples that demonstrate some design principles in action.

A [Au_55_(SR)_24_(PPh_3_)_6_]^3+^ NC (Au_55_, SR = SPh‐*p*‐Me) was synthesized via a halide‐free strategy [45], which differs from the previously proposed structure of Schmid's Au_55_(PPh_3_)_12_Cl_6_ [46]. The 55 atoms stack in an *fcc* structure with the facets protected by 24 *μ*_2_‐SR and 6 terminal phosphines. The Au_55_ NC showed higher FE toward CO within the voltage range of −0.5 to −0.9 V, with the best performance observed to be FE_CO_ = 94.1% at −0.6 V [45]. Moreover, the CO formation rate of Au_55_ reached 50.34 µL/min at −0.9 V, which is 2.4 and 1.96 times higher than those of the spherical and rod‐shaped Au_25_, respectively.

It is widely acknowledged that hydrogen is crucial during the electrocatalytic CO_2_‐to‐CO process, but a clear mechanism at the atomic level had been elusive until Au NCs with lattice hydrogen were achieved. In 2022, a heteroleptic [Au_22_H_3_(dppe)_3_(PPh_3_)_8_]^3+^ (Au_22_H_3_, dppe = 1,2‐bis(diphenylphosphino)ethane) NC was reported, which was composed of two Au_11_ units [47]. Importantly, three hydrides (H^–^) bridge the six uncoordinated gold atoms at the waist part of the NC, where two Au_11_ units fuse, creating the proposed active sites. The Au_22_H_3_ NC exhibited outstanding electrocatalytic activity for converting CO_2_ to CO, achieving a high FE of 92.7% at −0.6 V, along with a high mass activity of 134 A/g_Au_ and a TOF of 488 h^−1^ [47]. The performance significantly exceeded Au_11_ (FE_CO_ = 70.6%) and exhibited remarkable stability (over 10 h). DFT calculations revealed that the hydride ligands directly facilitate the formation of the key *COOH intermediate, lowering the energy barrier for CO production.

**FIGURE 2:**
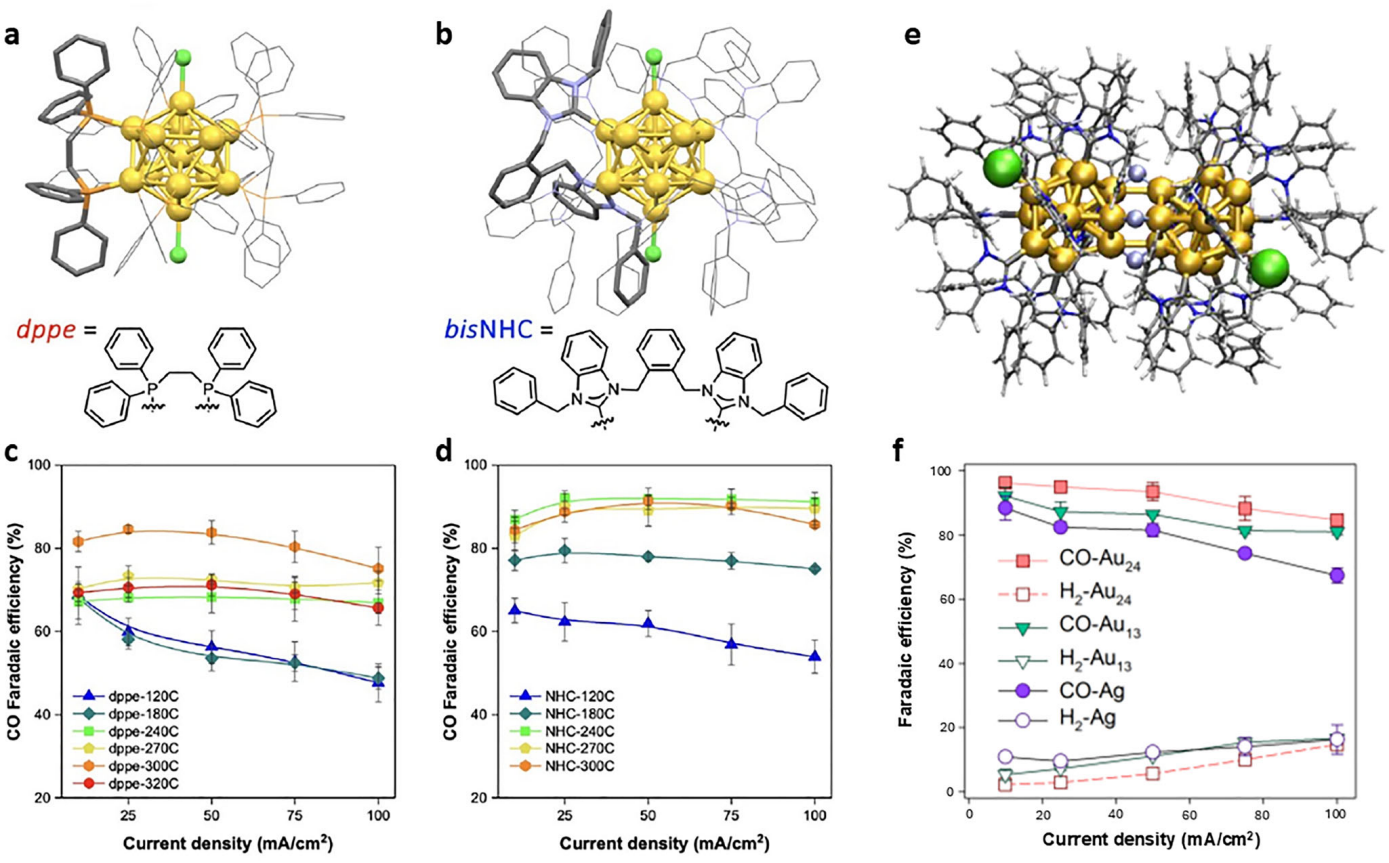
Crystal structures of (a) Au_13_‐dppe and (b) Au_13_‐NHC. FE_CO_ at different current densities of (c) Au_13_‐dppe and (d) Au_13_‐NHC after thermal treatment at different temperatures [49]. Copyright 2024, American Chemical Society. (e) Crystal structure of Au_24_H_3_‐NHC with three simulated hydrides. (f) CO and H_2_ Faradaic efficiencies at different current densities for CO_2_ reduction on Au Au_24_H_3_‐NHC [50]. Copyright 2022, American Chemical Society.

*N*‐heterocyclic carbenes (NHCs) are phosphine analogues and have attracted considerable attention in organometallic chemistry and recently in surface chemistry. NHC‐protected NCs were first synthesized with mono‐ or di‐substituted NHC ligands by introducing NHC into [Au_11_(PPh_3_)_8_Cl_2_]^+^ (Au_11_). In CO_2_RR, NHC‐functionalized NCs—especially the methyl‐substituted variant [Au_11_(PPh_3_)_7_(NHC‐Me)Cl_2_]⁺ (Au_11_‐NHC) exhibited a much higher FE_CO_ of 83% than Au_11_ (FE_CO_ = 35%) at −0.8 V with the highest current density and greatest mass activity among all the tested clusters [48]. This exceptional catalytic performance of Au_11_‐NHC is correlated with its enhanced thermal and structural stability [48]. A follow‐up work further explored the catalytic properties of [Au_13_(dppe)_5_Cl_2_]^3+^ (Au_13_‐dppe) and [Au_13_(*bis*NHC)_5_Cl_2_]^3+^ (Au_13_‐NHC) (Figure 2a,b). The Au_13_‐dppe required a high activation temperature (300°C) to reach a maximum FE_CO_ of 75.1% (Figure 2c), but the optimum thermal treatment for Au_13_‐NHC was much lower (240°C) and a high FE_CO_ of up to 91.2% was achieved, which maintained across a wide current density range up to 100 mA/cm^2^ over 100 h (Figure 2d) [49]. The enhanced performance is attributed to the stronger Au–C bonds and π–π interactions in Au_13_‐NHC, allowing only partial ligand removal during the catalyst activation. The heating process generates active sites while preserving the NC structure and preventing aggregation, forming highly active yet stable NC catalysts.

Based on the high stability of NHC‐protected Au NCs and the high reactivity of hydride species, NCs with both NHC and hydride become promising high‐performance catalysts. Thus, a [Au_24_(NHC)_14_Cl_2_H_3_]^3+^ (Au_24_H_3_‐NHC) NC with a bi‐icosahedral architecture bridged by three hydrides (Figure 2e) was applied to CO_2_RR, demonstrating an FE_CO_ greater than 90% across current densities ranging from 10 to 50 mA cm^−2^ with stable operation exceeding 100 h [50]. This NC also exhibited a high mass activity of 1360 A/g_Au_ @ 100 mA cm^−2^, significantly outperforming both Ag benchmarks and NHC‐protected Au_13_ (Figure 2f). DFT calculations indicated that the hydride not only contributes to structural integrity but also participates electronically, favoring the formation of key reaction intermediates by modulating the electronic state of the gold core.

**FIGURE 3:**
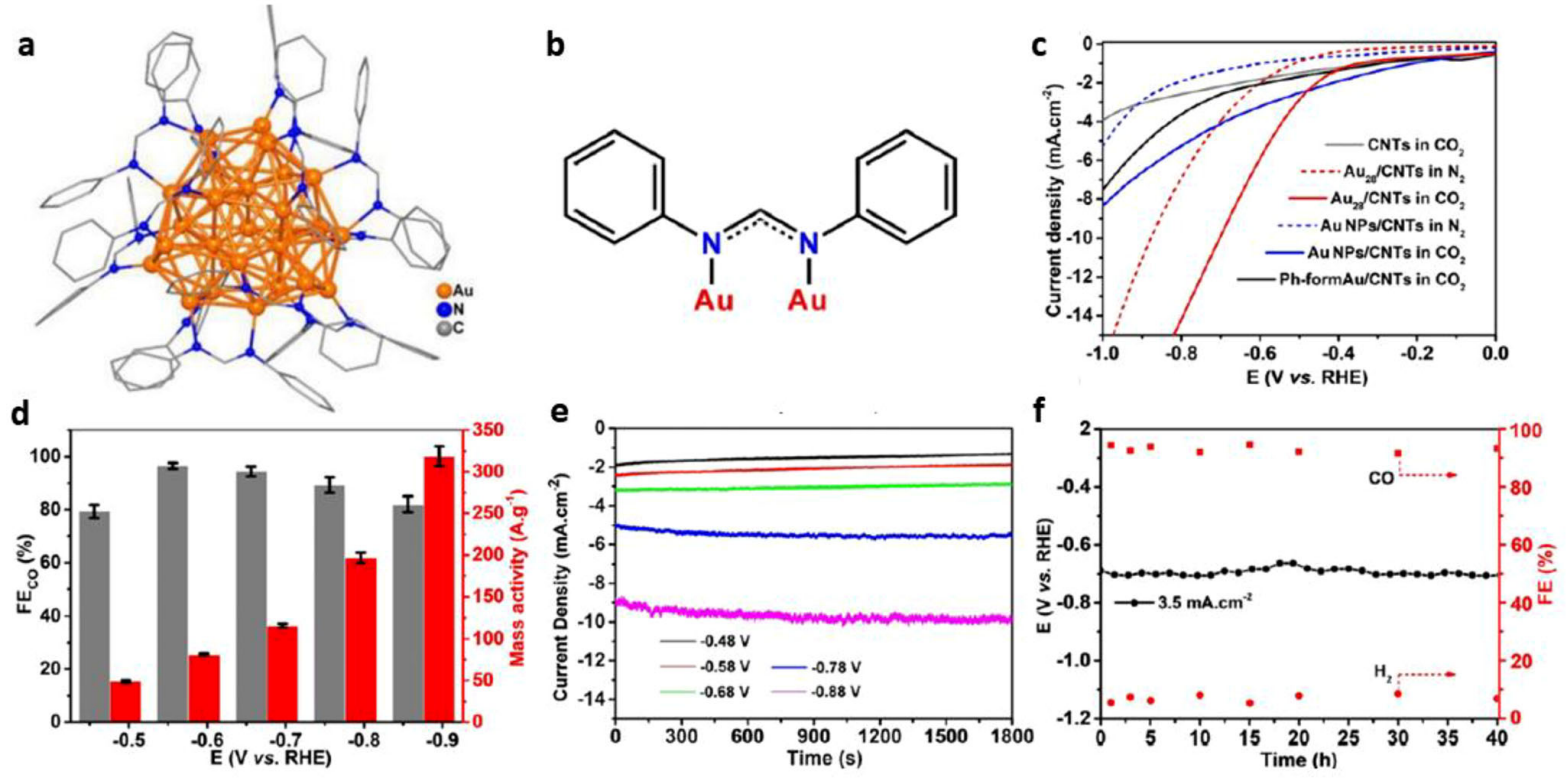
(a) Crystal structure of [Au_28_(Ph‐form)_12_]^2+^ and (b) the binding mode of Ph‐form on Au. (c) LSV of supported cluster catalysts, (d) Faradaic efficiency and mass activity of CO, and (e) current density of Au_28_/CNTs catalyst during CO_2_RR. (f) Long‐term electrochemical reaction at –3.5 mA/cm^2^ [51]. Copyright 2021, Wiley‐VCH.

In addition to NHC, *N*,*N*’–diphenylformamidinate (Ph‐form) has emerged as another ligand to synthesize highly stable gold NCs. The robustness of [Au_28_(Ph‐form)_12_]^2+^ (Au_28_) originates from its core structure of *T* symmetry fully passivated by 12 bridging formamidinate ligands through strong Au‐N bonds (Figure 3a,b), achieving both geometric and electronic shell closure [51]. Au_28_ loaded on hydroxylated multi‐walled carbon nanotubes (CNTs) achieved an FE_CO_ of 96.5% at −0.57 V, along with a CO formation rate of 318.7 A g^−1^ and a TOF of 1731 h^−1^ at −0.87 V (Figure 3c–e) [51]. The catalyst also maintained a steady potential around −0.69 V with FE_CO_ > 91% throughout a continuous 40‐h test at −3.5 mA cm^−2^ (Figure 3f).

Doping heteroatoms into Au NCs has been proven to be effective in improving various physicochemical properties, including the electrocatalytic activity, due to synergic effects [52]. CO production was detected at −0.34 V on Ag_25_, much higher than Au_25_ (−0.12 V). By replacing the original Ag_12_ shell with Au_12_ in the Ag_25_ structure, a [AuAg_12_@Au_12_(PET)_18_]^–^ (AuAg_12_@Au_12_) NC with gold occupying the catalytically critical surface sites exhibited dramatically enhanced CO_2_RR performance compared to Ag_25_ [53]. The new core−shell NC achieved a low onset overpotential close to that of Au_25_, with FE_CO_ approaching 80% to 92%. The Tafel slope of AuAg_12_@Au_12_, comparable to that of Au_25_, also indicated similar reaction kinetics between the two NCs. In a membrane electrode assembly (MEA) electrolyzer, AuAg_12_@Au_12_ delivered a current density of 339 mA/cm^2^ at a fuel cell potential of 2.66 V and remained stable for 24 h at 200 mA/cm^2^ with a cell potential of ∼2.13 V [53].

The open‐shell structure of [AuAg_26_(S–Adm)_18_S]^–^ (AuAg_26_) NC exposes four triangular facets of the kernel. When applied to electrocatalysis, AuAg_26_ demonstrated a Faradaic efficiency for CO of up to 98.6% at –0.97 V in a CO_2_‐saturated 1‐ethyl‐3‐methylimidazolium tetrafluoroborate ionic liquid containing water (EMIM‐BF_4_/H_2_O) as the electrolyte, which was much higher than that of Ag_25_ (∼55%) and Au_21_ (∼4%).[54]

To understand how the composition in structurally similar alloy NCs might impact the electrocatalytic properties, two M_15_ NCs—[Au_7_Ag_8_(SPh)_6_(P(Ph‐*p*‐OMe)_3_)_8_]^+^ (Au_7_Ag_8_) and [Au_13_Cu_2_(TBBT)_6_(P(Ph‐*p*‐Cl)_3_)_8_]^+^ (Au_13_Cu_2_)—were compared in CO_2_RR [55]. The Au_13_Cu_2_ demonstrated a FE_CO_ of 90.4% at −0.6 V, compared to 77.7% for Au_7_Ag_8_, highlighting the critical role of Cu doping in promoting CO production despite a lower electrochemically active surface area [55].

**FIGURE 4:**
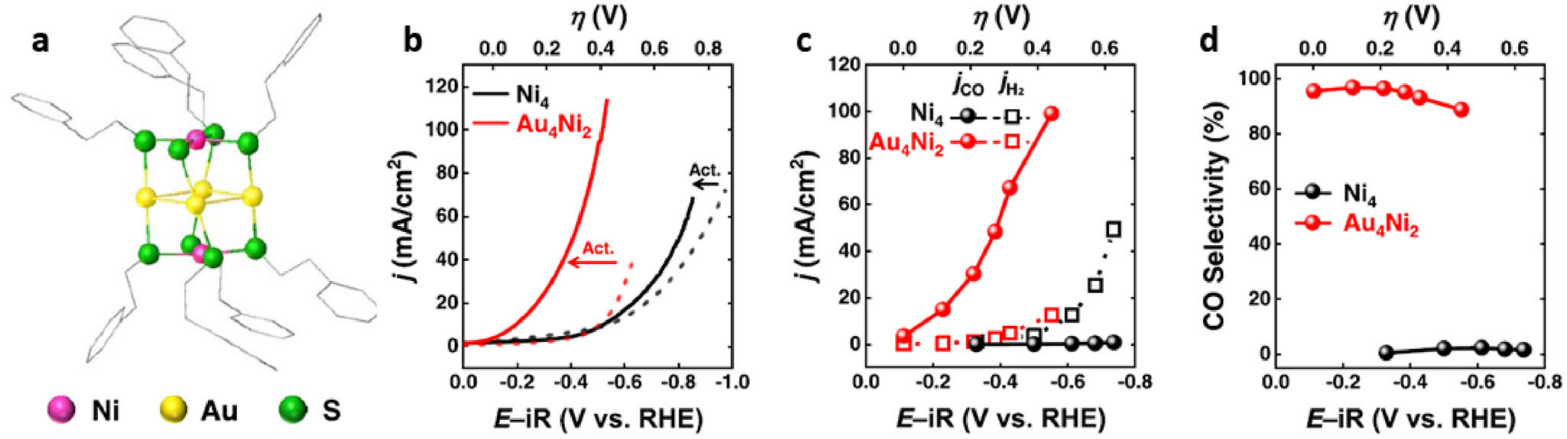
(a) Crystal structure of the Au_4_Ni_2_(PET)_8_. (b) LSV recorded at 50 mV/s on Ni_4_/GDE and Au_4_Ni_2_/GDE before (dashed lines) and after (solid lines) activation. (c) *j*_CO_ and *j*_H2_ acquired on Ni_4_/GDE and Au_4_Ni_2_/GDE, and (d) the corresponding CO selectivity as functions of cathodic potential. All panels reproduced from ref [54] with permission. Copyright 2023, American Chemical Society.

While Au‐based NCs exhibit extraordinary CO_2_‐to‐CO conversion, a large fraction of Au atoms is not involved in the CO_2_RR process. The low content of active species in gold‐based catalysts can be addressed by anchoring gold active sites onto a stable, nickel NC framework, thereby enhancing the utilization efficiency of gold atoms. During the electrochemical activation, Au_4_Ni_2_(PET)_8_, whose structure features a skewed octahedral core with four gold and two nickel atoms bridged by thiolate ligands (Figure 4a), undergoes the removal of approximately two thiolate ligands to form dethiolated Au sites as the active centers. After activation, Au_4_Ni_2_ immobilized on a gas‐diffusion electrode (GDE) achieved an FE exceeding 90% across a broad range from −0.1 V to −0.5 V, with a maximum of 97% at −0.23 V, and a *j*_CO_ of 100 mA cm^−2^ at −0.5 V (Figure 4b–d) [56]. It also showed a product formation rate of 206 mol_CO_ mol^−1^_NC_ s^−1^ at −0.32 V and exhibited high stability for 26 h (−0.76 V). Calculations revealed that the dethiolated Au sites are the active sites for the electroreduction of CO_2_ to CO, whereas the Ni sites become poisoned by the strongly adsorbed CO intermediates [56].

In addition to CO_2_RR, some NCs have also been applied to the NO_3_^–^ reduction reaction (NO_3_^–^RR). An active site “surgical” approach was adopted to manipulate active sites on NCs by leveraging ligand removal and site‐specific substitution. A carboranylthiolate/phosphine‐co‐protected [Ag_17_(C_4_B_20_H_20_S_4_)_6_(PPh_3_)_4_]^3−^ (Ag_17_P) was synthesized and reversibly transformed into [Ag_17_(C_4_B_20_H_20_S_4_)_6_]^3−^ (Ag_17_) with four exposed Ag sites through solvent‐induced phosphine dissociation [57]. Ag_17_P could be further site‐specifically substituted with Cu to form the isostructural [Ag_13_Cu_4_(C_4_B_20_H_20_S_4_)_6_]^3−^ (Ag_13_Cu_4_). In NO_3_^–^RR, both Ag_17_ and Ag_13_Cu_4_ exhibited greatly enhanced performance compared to ligand‐saturated Ag_17_P, with Ag_13_Cu_4_ achieving an FE of 90.4% for NH_3_ production and a total FE of 99.6%, attributed to optimized intermediate adsorption and accelerated kinetics via Cu doping [57].

By combining the two reaction processes of NO_3_^–^RR and CO_2_RR, nanocatalysts can further promote C–N coupling for urea synthesis. By replacing two phosphine ligands with carbene ligands, rod‐shaped [Au_13_Ag_12_(PPh_3_)_8_(BMIm)_2_I_8_]^+^ NCs (BMIm = 1,3‐bimethylimidazole) were synthesized and anchored onto NiFe layered double hydroxide (LDH) for electrocatalysis, exhibiting remarkable activity with a urea production rate of 29.5 mmol g_cat_^−1^h^−1^ and a Faradaic efficiency of 34% at −0.5 V (vs. RHE) [58]. Other Ag‐based alloy NCs, including Ag_14_Pd(PTFE)_6_(PPh_3_)_8_ and Ag_13_Au_5_(PTFE)_10_(dppp)_4_ (PTFE = pentafluorothiophenol, dppp = 1,3‐bis(diphenyphosphino)propane), also showed good performance in electrocatalytic urea synthesis, achieving maximum Faradaic efficiencies corresponding to production rates of 143.3 and 82.3 mg h^−1^ g_cat_^−1^, respectively [59].

The state‐of‐the‐art studies on electrochemical CO_2_RRs using atomically precise non–alkynyl‐protected nanoclusters as catalysts are summarized in Table 1.

**TABLE 1:** Electrochemical CO_2_ reduction reactions catalyzed on non–alkynyl‐protected nanoclusters.

NC catalyst	Reactor	Electrolyte	Product	Activity FE (vs. RHE)	Partial current density (mA cm^−2^) (vs. RHE)	Product formation rate/TON (vs. RHE)	Stability	Refs.
Au_25_(SC_6_H_13_)_18_	GDE	3 M KOH	CO	>90% @ −0.1 to −0.56 V (Sel. CO)	59 @ −0.56 V	128 mol_CO_ mol^−1^_NC_ s^−1^ @ –0.56 V	100 h @ −1.16 V 1 M KOH	[16]
Ag_38_(SC_6_H_13_)_24_					110 @ −0.56 V	186 mol_CO_ mol^−1^_NC_ s^−1^ @ –0.56 V	25 h @ −1.16 V 1 M KOH	
Au_144_(SC_6_H_13_)_60_					230 @ −0.56 V	590 mol_CO_ mol^−1^_NC_ s^−1^ @ –0.56 V		
Au_38_(PET)_24_Q	H‐Cell	0.5 M KHCO_3_	CO	∼100% @ > −0.8 V	∼35 @ −0.87 V	NA	NA	[17]
Au_144_(PET)_60_				∼100% @ > −0.8 V	∼26 @ −0.87 V			
Au_333_(PET)_79_				∼95% @ −0.8 V	∼22 @ −0.87 V			
Au_28_(TBBT)_20_				∼100% @ > −0.77 V	∼28 @ −0.87 V			
Au_36_(TBBT)_24_				∼100% @ > −0.77 V	∼25 @ −0.77 V			
Au_279_(TBBT)_84_				∼95% @ −0.6 V	∼16 @ −0.77 V			
Au_38_(PET)_24_T				∼97% @ −0.77 V	∼22 @ −0.87 V			
[Au_25_(*p*−MBA)_18_]^−^	H‐Cell	0.1 M KHCO_3_	CO	>75% @ −0.6 ∼ –1.4 V 90% @ –1.0 V	∼8 @ −0.9 V	∼70 s^−1^	NA	[37]
[Au_25_(*p*−MBA)_18‐x_(TCA)_x_]^−^				>90% @ −0.6 ∼ –1.4 V 98.6% @ –0.9 V	15.5 @ −0.9 V	138.8 s^−1^	18 h @ −0.9 V	
[Ag_25_(Capt)_18_]^−^	H‐Cell	0.5 M KHCO_3_	CO	45%–66% @ –0.5 V ∼ –0.7V	10.3 @ −0.82 V	NA	NA	[38]
[Ag_25_(IPBT)_18_]^−^	H‐Cell			>90% @ –0.5 V ∼ –0.7V	24 @ −0.82 V			
	MEA	0.1 M KHCO_3_		91.8% @ −3.4 V	240 @ −3.4 V		120 h @ –3.2 V	
spherical Au_25_	H‐Cell	0.5 M KHCO_3_	CO	73.7% @ −0.57 V	∼50 @ –0.87 V	33.3 µL/min @ –1.17 V	NA	[23]
rod‐shaped Au_25_				∼60% @ −0.87 V	∼15 @ −0.87 V	11.7 µL/min @ –1.17 V		
[Au_25_(PET)_18_]^–^	H‐Cell	0.1 M KHCO_3_	CO	∼100% @ −0.9 V	18.8 @ −0.9 V	∼980 mA mg^−1^ @ –1.2 V	NA	[25]
PdAu_24_(PET)_18_				∼100% @ −0.9 ∼ –1.2 V	20.3 @ −0.9 V	∼1770 mA mg^−1^ @ –1.2 V	6 h @ −0.8 V	
[Au_25_(SC_6_H_13_)_18_]^–^	H‐Cell	0.1 M KHCO_3_ + 0.4 M KCl	CO	>95% @ −0.6 ∼ –0.9 V (Sel.CO)	12 @ −0.7 V	7 mol_CO_ mol^−1^_NC_ s^−1^ @ –0.7 ∼ –1.0 V	NA	[39]
PtAu_24_(SC_6_H_13_)_18_				∼50% @ –0.6 ∼ –0.8 V	5 @ −0.9 V	7 mol_CO_ mol^−1^_NC_ s^−1^ @ –0.9 V		
Au_44_ (TBBT)_28_	H‐Cell	0.5 M KHCO_3_	CO	83% @ –0.57 V	1.6 @ −0.57 V	30.8 A g^−1^ @ –0.57 V	20 h @ −0.57 V	[41]
Au_47_Cd_2_(TBBT)_31_				96% @ −0.57 V	3.2 @ −0.57 V	55.6 A g^−1^ @ –0.57 V		
Au_23_(SR)_16_	H‐Cell	0.5 M KHCO_3_	CO	50%–65% @ –0.5 V ∼ –1.0V	∼21 @ −0.9 V	4.5 mol_CO_ mol^−1^_NC_ s^−1^ @ –1.0 V	NA	[26]
Au_19_Cd_2_(SR)_16_				90%–95% @ –0.5 V ∼ –0.9 V	∼42 @ −0.9 V	8.5 mol_CO_ mol^−1^_NC_ s^−1^ @ –1.0 V		
Au_25_(PET)_18_	H‐Cell	1 M KHCO_3_	CO	40%–70% @ –0.5 V ∼ ‐0.9V	7.0 @ −0.6 V	NA	10 h @ −0.7 V	[42]
Au_24_Cd(PET)_18_				90% ∼ 83% @ –0.5 V ∼ –0.9V	18.1 @ −0.6 V	68.5 A/g_metal_	24 h @ −0.7 V	
Au_19_Cd_3_(S‐tol)_18_				40%–62% @ –0.5 V ∼ –0.9 V	2.3 @ −0.6 V	NA		
Au_38_Cd_4_(d‐MBT)_30_				40%–58% @ –0.5 V ∼ –0.9 V	10.8 @ −0.6 V	NA	10 h @ −0.7 V	
[Au_55_(*p*−MBT)_24_(Ph_3_P)_6_]^3+^	H‐Cell	0.1 M KHCO_3_	CO	94.1% @ −0.6 V	50 @ −0.9 V	50.34 µL/min @ –0.9 V	4 h @ −0.6 V	[46]
[Au_22_H_3_(dppe)_3_(PPh_3_)_8_]^3+^	H‐Cell	0.5 M KHCO_3_	CO	92.7% @ −0.6 V	3.5 @ −0.6 V	134 A g^−1^_Au_ 488 h^−1^ @ –0.6 V	10 h @ −0.6 V	[47]
[Au_11_(dppe)_5_]^3+^				70.6% @ −0.6 V	2.2 @ −0.6 V	75.8 A g^−1^_Au_ 276 h^−1^ @ –0.6 V	NA	
[Au_11_(PPh_3_)_8_Cl_2_]^+^	H‐Cell	0.1 M KHCO_3_	CO	35% @ −0.8 V	∼3 @ −1.0 V	∼4 A g^−1^ @ −1.0 V	NA	[48]
[Au_11_(PPh_3_)_7_(NHC^iPr^)Cl_2_]^+^				80% @ −0.8 V	∼4.5 @ −1.0 V	∼12 A^−1^ @ −1.0 V		
[Au_11_(PPh_3_)_7_(NHC^Me^)Cl_2_]^+^				83% @ −0.8 V	∼8.5 @ −1.0 V	∼28 A g^−1^ @ −1.0 V		
[Au_13_(dppe)_5_Cl_2_]^3+^	MEA	0.1 M KHCO_3_	CO	75.1% @ −3.67 V	100	NA	NA	[49]
[Au_13_(*bis*NHC)_5_Cl_2_]^3^				91.2% @ −3.61 V	100	1824 A g^−1^_Au_ @ 100 mA cm^−2^	>100 h @ 100 mA cm^−2^	
[Au_24_(NHC)_14_Cl_2_H_3_]^3+^	MEA	0.1 M KHCO_3_	CO	>90% @ 10 ∼ 50 mA.cm^−2^	100 @ ∼3.00V	1360 A g^−1^_Au_ @ 100 mA cm^−2^	>100 h @ 100 mA cm^−2^	[50]
[Au_28_(Ph‐form)_12_]^2+^	H‐Cell	0.5 M KHCO_3_	CO	96.5% @ −0.57 V	∼10 @ −0.88 V	1731 h^−1^ 318.7 A g^−1^ @ –0.87 V	40 h @ −3.5 mA cm^−2^	[51]
Ag_25_(SPhMe_2_)_18_	GDE	1 M KOH	CO	> 90% @ –0.5 V ∼ –0.6V	100 @ −0.6 V	NA	NA	[53]
Au_25_(PET)_18_				> 90% @ –0.1 V ∼ –0.3V	120 @ −0.3 V			
AuAg_12_@Au_12_(PET)_18_				80%–92% @ –0.1 V ∼ –0.3V	∼120 @ −0.3 V			
	AEM			90% @ −2.29V	339 @ −2.66 V		24 h @ 200 mA cm^−2^	
[AuAg_26_(S−Adm)_18_S]^−^	H‐Cell	EMIM‐BF_4_ /H_2_O v/ v = 7:1	CO	98.6% @ −0.97 V	9 @ −0.97 V	NA	11 h @ –0.97V	[54]
[Au_7_Ag_8_(SPh)_6_ (P(Ph‐*p‐*OMe)_3_)_8_]^+^	GDE	1 M KOH	CO	77.7%@‐0.6 V 61.8%@‐1.2 V	25–110 @ −0.6 ∼ –1.2 V	NA	NA	[55]
[Au_13_Cu_2_(TBBT)_6_ (P(Ph‐*p*‐Cl)_3_)_8_]^+^				90.4%@ −0.6 V 59.2%@‐1.2 V	58–150 @ −0.6 ∼ –1.2 V			
Au_4_Ni_2_(PET)_8_	GDE	1 M KOH	CO	>90% @ −0.1 ∼ –0.5 V 97% @ –0.23V	100 @ −0.5 V	206 mol_CO_ mol^−1^_NC_ s^−1^ @ –0.32 V	26 h @ –0.76 V	[56]

## 3 Cu‐Based Non–Alkynyl‐Protected Nanoclusters for Electrocatalysis

Copper is unique among CO_2_ reduction catalysts in its ability to produce hydrocarbons at significant current densities. Other metals show the contrast: those with high hydrogen overvoltage and negligible CO adsorption (Hg, Cd, Pb, Tl, In, and Sn) reduce CO_2_ efficiently but predominantly yield formate, indicating no C–C coupling. Metals with low hydrogen overvoltage (Pt, Ni, Fe, and Ti) reduce CO_2_ to strongly adsorbed CO but are poor at converting it further, leading instead to hydrogen as the dominant product [60]. Only metals with intermediate hydrogen overvoltage and weak CO binding enable C–O bond cleavage and CO desorption. In this context, Au, Ag, and Zn primarily produce CO, whereas Cu is able to reduce CO further, generating multicarbon products [61]. Thus, Cu is one of the few heterogeneous catalysts to show a propensity to produce valuable hydrocarbons and alcohols with decent efficiency in CO_2_RR [62]. The following cases illustrate how structural precision and ligand engineering in Cu‐based NCs enable control over reaction pathways promoting the generation of products other than CO in CO_2_RR.

Conventional copper catalysts are critically limited by the high overpotentials required for hydrocarbon production during CO_2_RR. To fundamentally comprehend this mechanism, a Cu_32_H_20_(S_2_P(O^i^Pr)_2_)_12_ (Cu_32_) NC was used as a model system to explore a lattice‐hydride mechanism, in which the anionic hydrides in the cluster lattice are directly involved in the CO_2_RR [63]. The electrocatalytic tests revealed an exceptional selectivity for HCOOH production at low overpotentials, achieving the highest FE_HCOOH_ of 89% at −0.52 V. However, such selectivity dramatically reversed at < −0.72 V, giving rise to HER. DFT calculations revealed that the negatively charged lattice hydrides directly transfer to CO_2_ to form HCOOH via a low‐energy pathway, while the competing pathways to CO and HER require much higher kinetic barriers [63].

**FIGURE 5:**
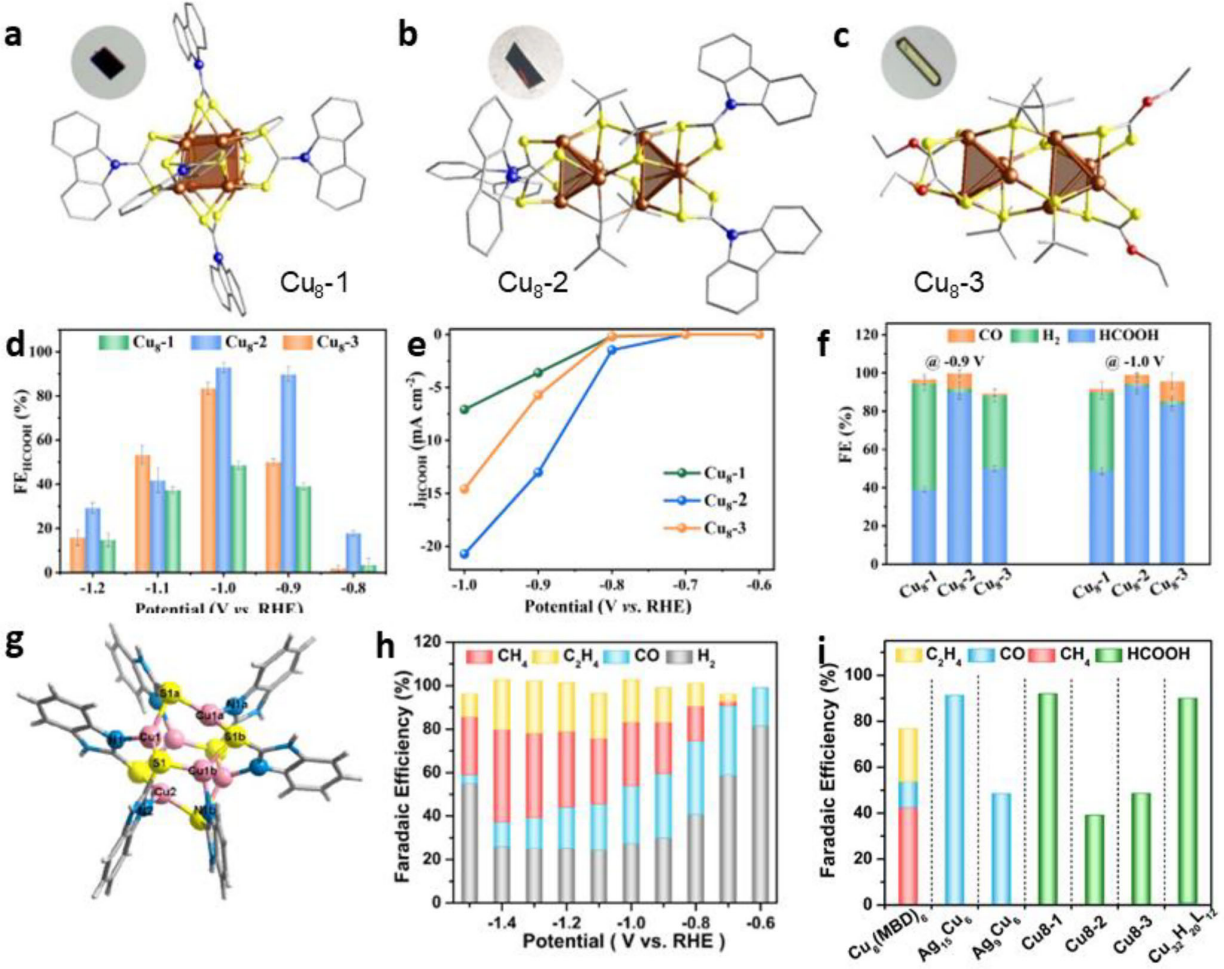
Crystal structures of (a) Cu_8_‐1, (b) Cu_8_‐2, and (c) Cu_8_‐3. (d) FE_HCOOH_ of Cu_8_‐1, Cu_8_‐2, and Cu_8_‐3 at different applied potentials. (e) FEs for HCOOH, CO, and H_2_ of Cu_8_ isomers at different applied potentials. (f) *j*_HCOOH_ of Cu_8_ isomer catalysts [64]. Copyright 2022, Wiley‐VCH. (g) Crystal structure of Cu_6_(MBD)_6_. (h) Faradaic efficiencies for CH_4_, C_2_H_4_, CO, and H_2_ at different potentials of Cu_6_(MBD)_6_, and (i) comparison of the CO_2_RR performances of different Cu NCs [66]. Copyright 2023, Wiley‐VCH.

The impact of isomers on the CO_2_RR was investigated using three Cu_8_ NC isomers, including Cu_8_H(L_1_)_6_PF_6_ (Cu_8_‐1) with a twisted cubic Cu_8_ core, Cu_8_(S*^t^* Bu)_4_(L_1_)_4_ (Cu_8_‐2) and Cu_8_(S*^t^* Bu)_4_(L_2_)_4_ (Cu_8_‐3) with ditetrahedral Cu_8_ cores (L_1_ = 9H‐carbazole‐9‐carbodithioate and L_2_ = *o*‐ethyl carbonodithiolate), which were synthesized via a ligand‐controlled strategy (Figure 5a–c) [64]. These catalysts confirmed that the CO_2_RR activity was dominated by the core architecture [64]. Cu_8_‐2 exhibited the best electrocatalytic performance, achieving a FE_HCOOH_ of up to 92% at −1.0 V, which was more than twice that of Cu_8_‐1 (48.6%) and slightly better than Cu_8_‐3 (∼85%). The *j*_HCOOH_ of Cu_8_‐2 (∼24 mA cm^−2^) was also significantly higher than those of Cu_8_‐1 and Cu_8_‐3 (∼7.5 and ∼15 mA cm^−2^, respectively) at −1.0 V (Figure 5d–f). Cu_8_‐2 maintained its stability for 8 h without current attenuation.

A systematic study on four Cu‐based NCs—[MCu_24_H_22_(PR_3_)_12_]⁺ (M = Au or Cu, R = Ph or *p*‐FPh) also showed that both ligand and doping effects significantly influence the catalytic activity of Cu NCs [65]. Electrocatalytic testing revealed that central Au‐doped NCs significantly suppressed HER and enhanced CO_2_ reduction, with AuCu_24_‐PPh_3_ achieving a FE_CO_ of 45.6% at −1.0 V, whereas homometallic Cu_25_ predominantly produced H_2_. Moreover, the electron‐withdrawing ligands shifted the selectivity toward HCOOH, resulting in an FE_HCOOH_ of 30.6% for AuCu_24_‐P(*p*‐FPh)_3_ [65].

The CO_2_RR activity of Cu NCs can also be improved by atomic‐level tuning of ligands and coordination structures to enable C–C coupling beyond C_1_, converting CO_2_ to more valuable hydrocarbon products. A novel Cu_6_(MBD)_6_ NC (MBD = 2‐mercaptobenzimidazole) was designed using a ligand that creates symmetry‐broken Cu–S_2_N_1_ active sites (Figure 5g) [66]. Electrocatalytic evaluation revealed that this NC achieved a total hydrocarbon FE of 65.5% at −1.4 V, comprising 42.5% CH_4_ and 23% C_2_H_4_, with a high partial current density of −183.4 mA/cm^2^ (Figure 5h,i), while completely suppressing HCOOH production [66]. Calculations suggested that the asymmetric Cu–S_2_N_1_ sites favor CO_2_ binding via the carbon atom, lowering the energy barrier for *COOH formation and promoting stabilization of the CO intermediate, which subsequently underwent hydrogenation and C–C coupling to yield CH_4_ and C_2_H_4_.

The underlying mechanism involving lattice hydrogens on Cu NCs was further revealed by designing a bimetallic [AuCu_24_H_22_(dppp)_6_]⁺ (AuCu_24_‐dppp, dppp = 1,3‐bis(diphenyphosphino)propane) NC [67]. The presence of surface cavities that fully expose triangular Cu_3_H_3_ units is critical for C_2+_ generation, in stark contrast to a similar NC capped by triphenylphosphine (AuCu_24_‐tpp), where the active sites are buried. AuCu_24_‐dppp exclusively favored C_2+_ products with a total FE exceeding 50%, achieving FE_C2H4_ = 41.9% and FE_EtOH_ = 12.6%, respectively, at −1.5 V, with a current density of 96.5 mA cm^−2^ at −1.5 V. In contrast, [AuCu_24_H_22_(tpp)_12_]⁺ (AuCu_24_‐tpp) was highly selective for C_1_, with FE_CO_ of ∼45% at −0.8 V and FE_HCOOH_ of 39.6% at −0.9 V. Integration of in situ infrared spectroscopy, isotope labeling and DFT calculations showed that lattice hydrides in the exposed Cu_3_H_3_ units are responsible for lowering the energy barrier for the key *COCOH intermediate to form, enabling further C–C coupling [67].

The literature on non–alkynyl‐protected Cu‐based NCs is summarized in Table 2.

**TABLE 2:** Electrochemical CO_2_ reduction reactions catalyzed on non–alkynyl‐protected Cu‐based nanoclusters.

NC catalyst	Reactor	Electrolyte	Product	Activity FE (vs. RHE)	Partial current density (mA cm^−2^) (vs. RHE)	Product formation rate/TON (vs. RHE)	Stability	Refs.
Cu_32_H_20_(S_2_P(O*^i^* Pr)_2_)_12_	H‐Cell	0.1 M KHCO_3_ + 0.4 M KCl	HCO_2_H	89% @ −0.52 V	∼25 @ −0.52 V	NA	3 h @ −0.52 V	[63]
Cu_8_(H)(L_1_)_6_PF_6_	H‐Cell	0.5 M KHCO_3_	HCO_2_H	48.6% @ −1.0 V (HCO_2_H)	∼7.5 @ −1.0 V	8 mol_HCOOH_ mol^−1^_NC_ min^−1^ @ –1.0 V	8 h @ −1.0 V	[64]
Cu_8_(S*^t^* Bu)_4_(L_1_)_4_				92% @ –1.0 V (HCO_2_H)	∼21 @ −1.0 V	17.5 mol_HCOOH_ mol^−1^_NC_ min^−1^ @ –1.0 V		
Cu_8_(S*^t^* Bu)_4_(L_2_)_4_				∼85% @ −1.0 V (HCO_2_H)	∼15 @ −1.0 V	∼11 mol_HCOOH_ mol^−1^_NC_ min^−1^ @ –1.0 V		
[Cu_25_H_22_(PPh_3_)_12_]^+^	H‐Cell	0.5 M KHCO_3_	CO HCO_2_H	0–3.7% @ −0.8 ∼ −1.0 V (CO)	0.25–1.1 @ −0.8 V ∼ −1.2 V (HCOOH)	NA	12 h @ −0.8V	[65]
				6–10% @ −0.8 ∼ −1.0 V (HCO_2_H)				
[AuCu_24_H_22_(PPh_3_)_12_]^+^				45.6% @ −1.0 V (CO)	2 ∼ 8 @ −0.8 V ∼ −1.2 V (CO)			
				5%–12% @ −0.8 ∼ −1.2 V (HCO_2_H)				
[Cu_25_H_22_(P(*p*‐FPh)_3_)_12_]^+^				2%–8.2% @ −0.8∼ −1.2 V (CO)	3.2 @ −1.1 V			
				20.3% @ –0.8 V (HCO_2_H)				
[AuCu_24_H_22_(P(*p*‐FPh)_3_)_12_]^+^				11.8%–20.6% @ –0.8 V ∼ –1.2 V (CO)	2.6 @ −1.1 V			
				30.6% @ −0.8 V (HCO_2_H)				
Cu_6_(MBD)_6_	GDE	1 M KOH	CO	31.8% @ −0.7 V (CO)	48 @ −1.2 V (CO)	NA	NA	[66]
			CH_4_	42.5% @ −1.4 V (CH_4_)	119 @ −1.4 V (CH_4_)			
			C_2_H_4_	23%@ −1.4 V (C_2_H_4_)	64.4 @ −1.4 V (C_2_H_4_)			
[AuCu_24_H_22_(dppp)_6_]^+^			C_2_H_4_ EtOH	∼80% @ −0.8 V 38 ∼ 80% @ –0.8 ∼1.3 V (main CO)	96.5 @ −1.5 V (C_2_H_4_) 21.9 @ −1.6 V (EtOH)		100 h @ −1.5 V	
				41.9% @ −1.5 V 35%–42% @ –1.3 ∼ –1.7 V (main C_2_H_4_)				
	GDE	1 M KOH	CO	∼45% @ –0.8 V (CO)	20.7 @ −1.3 V (C_2_H_4_) 9.5 @ −1.6 V (EtOH)	NA		[67]
[AuCu_24_H_22_(PPh_3_)_12_]^+^			HCO_2_H	39.6% @ −0.9 V (HCO_2_H)			NA	
				15.3% @ −1.3 V (CH_4_)				
			CH_4_					
				6.4% @ −1.4V(EtOH)				

## 4 Alkynyl‐Protected Nanoclusters for Electrocatalysis

Alkynyl‐protected coinage NCs with atomic precision are a unique category due to the unsaturated triple bond in alkynyl ligands, bringing new interfacial structures and properties [68]. A distinct feature of alkynyl ligands is that their C≡C bond can anchor on a metal surface via both σ and π bonding, being much different from thiolates or phosphines. Such a flat arrangement of alkynyl ligands on NCs can reduce the ligand‐to‐metal ratio due to steric effects, thereby exposing more metal active sites for catalysis. In this section, we highlight recent works on alkynyl‐protected coinage NCs, showing how the surface chemistry and heterometal incorporation enhance catalytic performance across various electroreduction reactions.

Alkynyl‐protected Ag_32_(C≡CAr^F^)_24_ (Ag_32_, HC≡CAr^F^ = 3,5‐bis(trifluoromethyl)phenylacetylene) exhibited greatly suppressed HER and achieved the highest FE_CO_ of up to 96.4% at −0.8 V [69]. By contrast, [Ag_32_(SR)_24_(dppe)_5_]^2−^ of the same atomicity but different ligands showed a maximum FE_CO_ of only 57% but FE_H2_> 50% in the potential range of −0.7 to −1.0 V [69]. Another homoleptic [Ag_15_(C≡C*^t^* Bu)_12_]^+^ (Ag_15_) NC with a body‐centered cubic (*bcc*) Ag@Ag_8_@Ag_6_ core was found to convert CO_2_ into CO with a high FE of 95.0% at −0.6 V, a maximal TOF of 6.37 s^−1^ at −1.1 V, and long‐term stability at −0.75 V, for 10‐h‐electrolysis [70].

The 15‐metal‐atom NC template was further used to systematically study the composition effects on CO_2_RR. Three NCs, including [Au_7_Ag_8_(C≡C*^t^* Bu)_12_]^+^ (Au_7_Ag_8_), [Ag_9_Cu_6_(C≡C*^t^* Bu)_12_]^+^ (Ag_9_Cu_6_) and [Au_2_Cu_5_Ag_8_(C≡C*^t^* Bu)_12_]^+^ (Au_2_Cu_5_Ag_8_) were successfully synthesized [71]. Au_7_Ag_8_ demonstrated the highest selectivity of FE_CO_ = 98.1% (FE_CO_ > 90% at −0.49 to −1.19 V) and partial current density *j*_CO_ = 155 mA cm^−2^ at –0.49 V. On the other hand, CO and HCOOH were the main products for Ag_9_Cu_6_ and Au_2_Ag_8_Cu_5_ at more negative potentials, with the highest FE_HCOOH_ of 47.0% at −1.19 V and 28.3% at −0.99 V, respectively. At even higher potentials, CO became the predominant product for both Ag_9_Cu_6_ and Au_2_Ag_8_Cu_5_, showing FE_CO_ >95% [71].

**FIGURE 6:**
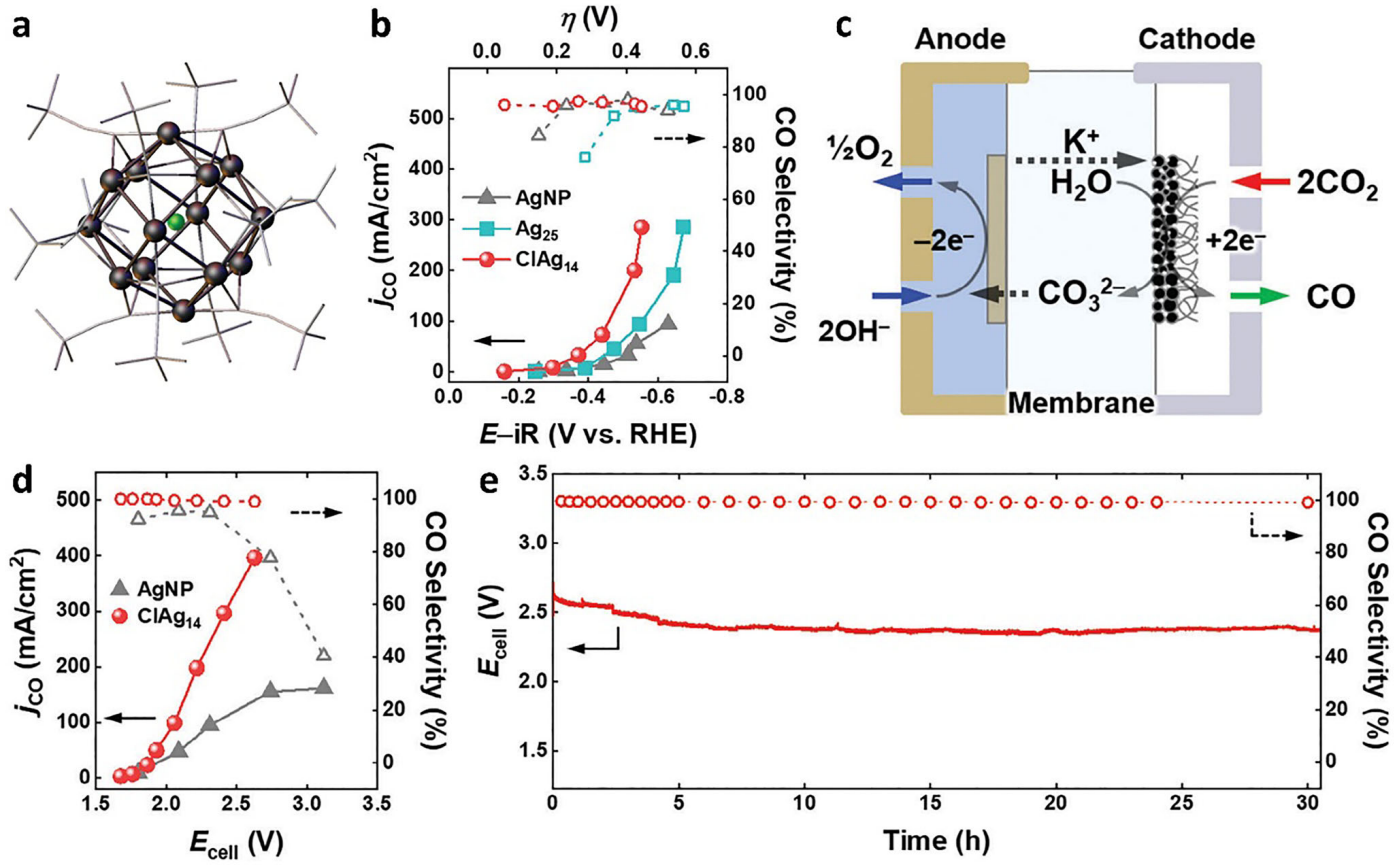
(a) Crystal structure of ClAg_14_ and (b) *j_CO_* and CO selectivity of various Ag catalysts employed in GDE. (c) Schematic of a MEA electrochemical cell, (d) *j_CO_* and CO selectivity of ClAg_14_ and Ag‐NP, and (e) long‐term CO_2_‐to‐CO electrolysis performed on ClAg_14_/MEA [72]. Copyright 2023, The Authors.

[ClAg_14_(C≡C*^t^* Bu)_12_]^+^ (ClAg_14_) NC was obtained when replacing the central Ag in Ag_15_ with a Cl atom (Figure 6a) [72]. The ClAg_14_ exhibited CO_2_RR activity for reduction to CO in both H‐cell and GDE systems. In the H‐cell, FE_CO_ reached up to 87% at −0.75 V, and the GDE employing 1 M KOH showed CO selectivity > 95% and a partial current density of CO of 285 mA cm^−2^ at −0.44 V (Figure 6b). Subsequently, a MEA electrochemical cell (Figure 6c) was used to evaluate the CO_2_RR activity under industrial conditions, in which ClAg_14_ achieved industrially significant electroreduction of CO_2_ to CO at a conversion efficiency of 51%, with a catalytic activity exceeding 1400 A g^−1^ at 400 mA cm^−2^ (Figure 6d), and good electrochemical stability for 30 h at 200 mA cm^−2^ (Figure 6e) [72].

**FIGURE 7:**
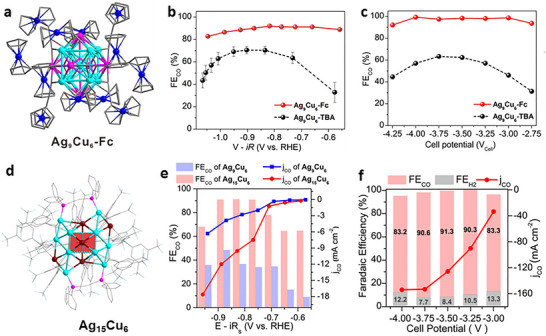
(a) Crystal structure of Ag_9_Cu_6_‐Fc. FE_CO_ of Ag_9_Cu_6_‐Fc and Ag_9_Cu_6_ at different applied potentials in (b) the H‐cell and (c) the MEA device [73]. Copyright 2024 Wiley‐VCH GmbH. (d) Crystal structure of Ag_15_Cu_6_. (e) FE_CO_ and *j_CO_* of Ag_15_Cu_6_ and Ag_9_Cu_6_ at various applied potentials in H‐cell, and (f) FE_CO_, FE_H2_, and *j_CO_* of Ag_15_Cu_6_ at different cell potentials in MEA cell [74]. Copyright 2023, American Chemical Society.

The CO_2_RR activity of the Ag_9_Cu_6_ NC could be improved through a ligand engineering strategy by integrating the NC with organometallic compounds to obtain a ferrocenylethyne‐protected [Ag_9_Cu_6_(C≡CFc)_12_]^+^ (Ag_9_Cu_6_‐Fc) NC (Figure 7a). When employing an H‐cell reactor, the FE_CO_ of Ag_9_Cu_6_‐Fc consistently exceeded 83% at the tested potential window, reaching a maximum of 92% whereas the Ag_9_Cu_6_ protected by *tert*‐butylacetylene (TBA) only exhibited a maximum FE_CO_ of ∼70% (Figure 7b) [73]. When a MEA cell was employed, FE_CO_ of Ag_9_Cu_6_‐Fc approached 100% from −4.25 V to −2.75 V, with a maximum *j*_CO_ of 680 mA cm^−2^, achieving industrial‐level performance standards (Figure 7c). Ag_9_Cu_6_‐Fc also showed excellent electrochemical stability (nearly undiminished performance over 200 h at a cell voltage of 3.00 V. In situ characterization and DFT demonstrated that ferrocene functionalization facilitated electron transfer and created a unique local chemical environment at the AgCu active site, leading to markedly improved catalytic activity and selectivity.

Other types of ligands (e.g., phosphines and halogen) have been applied together with alkynyl ligands to expand the library of NCs of atomic precision. When the *bcc* Ag_11_Cu_4_ core was further capped by 2 Cu atoms, 2 Ag_2_dppe motifs, and 18 alkynyl ligands, a [Ag_15_Cu_6_(C≡CAr^F^)_18_(dppe)_2_]^−^ (Ag_15_Cu_6_, Figure 7d) NC was obtained and tested for CO_2_RR [74]. In an H‐cell, Ag_15_Cu_6_ supported on carbon showed high FE_CO_ (> 85%) in the potential region from −0.72 to −0.87 V with the highest FE_CO_ up to 91.3% at −0.81 V. By contrast, the highest FE_CO_ of the *bcc* Ag_9_Cu_6_ NC without additional surface structure was only 48.5% at −0.89 V (Figure 7e). When evaluated in a MEA cell, Ag_15_Cu_6_ also exhibited FE_CO_ values above 83% in the range −3.00 to −4.00 V, with the highest FE_CO_ reaching 91.3% at −3.50 V and *j*_CO_ up to −154 mA cm^−2^ at −4.00 V (Figure 7f) [74].

Another alkynyl and phosphine co‐protected Ag_14_Cu_2_(C≡CAr^F^)_14_(PPh_3_)_4_ (Ag_14_Cu_2_) NC showed an FE_CO_ of 83.71% and a current density of 92.65 mA cm^−2^ at −1.6 V. Ag_14_Cu_2_ was also robust over 10 h of continuous operation in a GDE, as no significant decay in current density, and FE_CO_ was observed [75].

When halogen ligands were further involved, an Ag_19_Cu_2_(C≡CAr^F^)_12_(PPh_3_)_6_Cl_6_ (Ag_19_Cu_2_) NC was obtained, exhibiting a high FE_CO_ of 95.26%, a high current density of 257.2 mA cm^−2^ at −1.3 V, and high stability throughout the 14 h of continuous electrolysis [76]. The crystal structure of [Au_15_Cu_4_(C≡CAr^F^)(dppm)_6_Cl_4_]^2+^ (Au_15_Cu_4_, dppm = bis(diphenylphosphino)methane) revealed that the Au and Cu atoms preferentially coordinate with phosphines and chlorides, respectively. Upon Cu doping, Au_15_Cu_4_ measured in a MEA cell showed an FE_CO_ that consistently exceeded 90% from −3.75 V to −2.75 V, with *j*_CO_ reaching 413 mA cm^−2^ at 3.75 V, two‐fold higher than that of [Au_18_(dppm)_6_Br_4_]^2+^ [77].

Ag_4+_ *_x_* Au_40‐_ *_x_* (C_10_H_9_)_28_ (Ag_4+_ *_x_* Au_40‐_ *_x_*, C_10_H_9_ = 1‐ethynyl‐2,4‐dimethylbenzene) NC was prepared using Au_44_(C_10_H_9_)_28_ (Au_44_) as the template for Ag doping. Four Ag atoms are fixed in the surface staples, while six positions in the core have partial occupation by Au or Ag. Ag doping significantly disturbed the electronic configuration, and thereby Ag_4+_ *_x_* Au_40‐_ *_x_* exhibited a maximum FE_CO_ of 98% at −0.5 V in CO_2_RR, significantly higher than Au_44_ (FE_CO_ = 76% at −0.6 V) [78].

**FIGURE 8:**
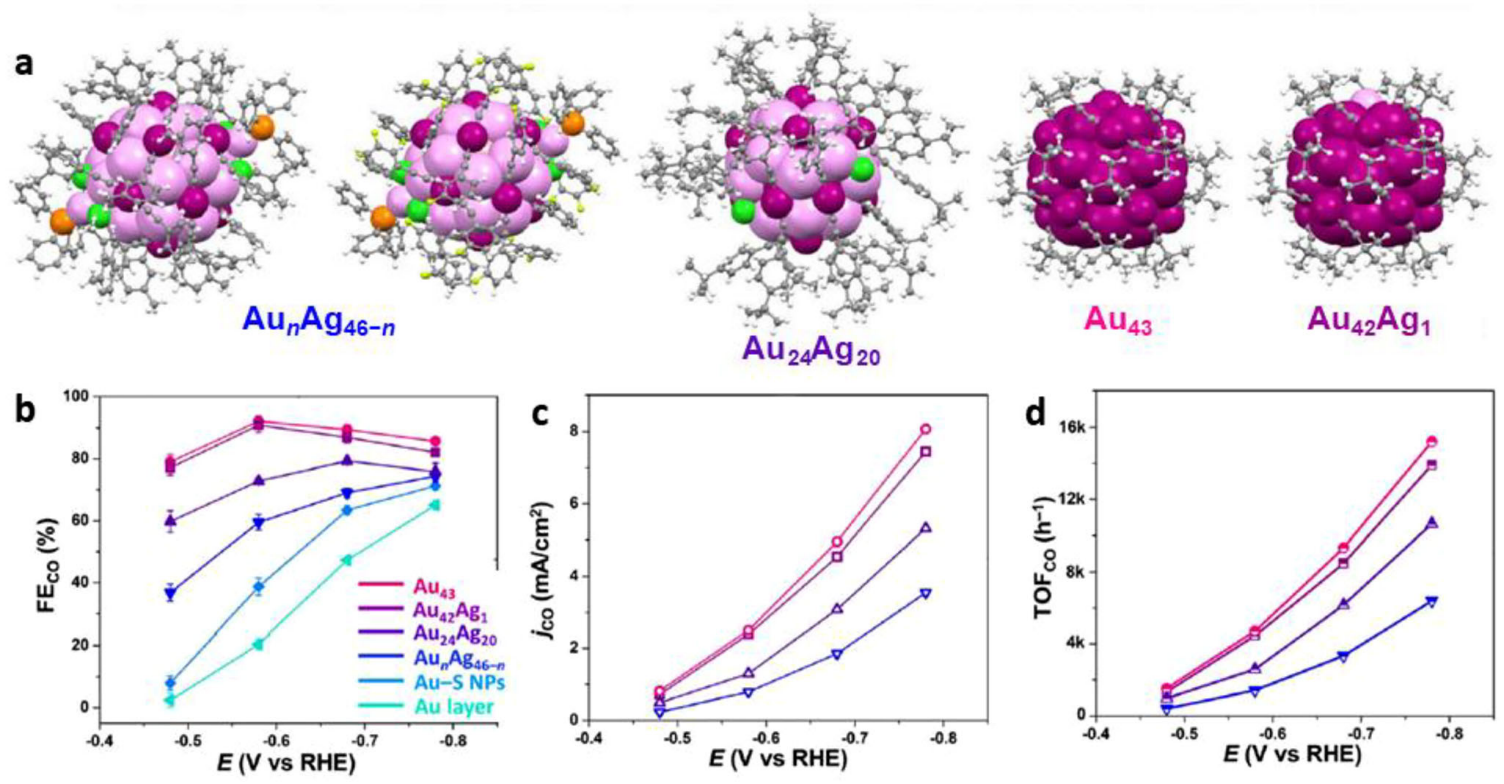
(a) Crystal structures of Au*_n_* Ag_46−_ *_n_*, Au_24_Ag_20_, Au_43,_ and Au_42_Ag_1_. (b) FE_CO_, (c) *j*_CO,_ and (d) TOF_CO_ for the series of NC‐based catalysts [79]. Copyright 2023, The Author(s).

Correlated NC series can serve as an effective model for CO_2_RR investigation [12]. Generally, the catalytic performance is often affected by many factors such as the surface/interface structure, electronic structure, ligand type, and metal core size, making it challenging to fully understand the structure–activity relationship [11]. A series of alkynyl‐protected NCs, including Au_n_Ag_46−n_(C≡CR)_24_Cl_4_(PPh_3_)_2_ (Au_n_Ag_46−n_, R = Ph–*m*–F or Ph–*m*–CH_3_), Au_24_Ag_20_(C≡CPh*^t^* Bu)_24_Cl_2_ (Au_24_Ag_20_) and double open‐shelled Au_43_(C≡C*^t^* Bu)_20_ (Au_43_) and Au_42_Ag(C≡C*^t^* Bu)_20_ (Au_42_Ag) were synthesized, with similar sizes (M_46_, M_44_ and M_43_, M = Ag/Au) and structures (icosahedral) but different ligand packing densities (Figure 8a) [80]. The approach aimed to provide a general picture linking the number of accessible metal active sites on the catalyst to CO_2_RR activity. The trend showed that FE_CO_ decreases as the ligand packing density on the cluster surface increases (Figure 8b, c) [79]. Specifically, NCs with lower surface ligand packing densities, Au_43_ and Au_42_Ag_1_ (accessible metal atoms *N* = 16), exhibited the highest FE_CO_, achieving 92.1% and 90.9% at −0.57 V, respectively, and remaining above 80% across the tested potential range. In contrast, Au_24_Ag_20_ (*N* = 12) and Au_n_Ag_46−n_ (*N* = 5) showed much lower FE_CO_ of 72.9% and 59.5%, respectively, under identical conditions. The CO TOFs for these catalysts were 4718, 4458, 2597, and 1427 h^−1^, respectively (Figure 8d). The influence of accessible metal sites was further applied to other Au–alkynyl NCs, including Au_44_(C≡CR)_28_ (*N* = 12, FE_CO_ = 74.5%), Au_36_(C≡CR)_24_ (*N* = 9, FE_CO_ = 69.7%), and Au_23_(C≡CR)_15_ (*N* = 6, FE_CO_ = 66.7%). The findings demonstrated that the number of accessible metal sites of NCs can be used as a simple indicator for evaluating the potential of atomically precise NCs for electrocatalysis [79].

To investigate how a subtle surface tailoring of NCs can significantly influence their physicochemical properties, another work pertains to NCs with an identical metal kernel but different surface ligands for CO_2_RR [36]. Au_28_(C_2_B_10_H_11_S)_12_(tht)_4_Cl_4_ (denoted Au_28_‐S) co‐protected by carboranylthiolate, tetrahydrothiophene (tht), and chloride, maintained the same metallic core structure as the alkynyl‐protected Au_28_(C_4_B_10_H_11_)_12_(tht)_8_^3+^ (Au_28_‐C). During CO_2_RR, Au_28_‐S exhibited superior catalytic activity for CO_2_‐to‐CO conversion, achieving 98.5% FE_CO_ at −0.9 V with a *j*_CO_ nearly two‐fold higher than that of Au_28_‐C (7.6 mA cm^−2^ vs. 3.8 mA cm^−2^) [82].

**FIGURE 9:**
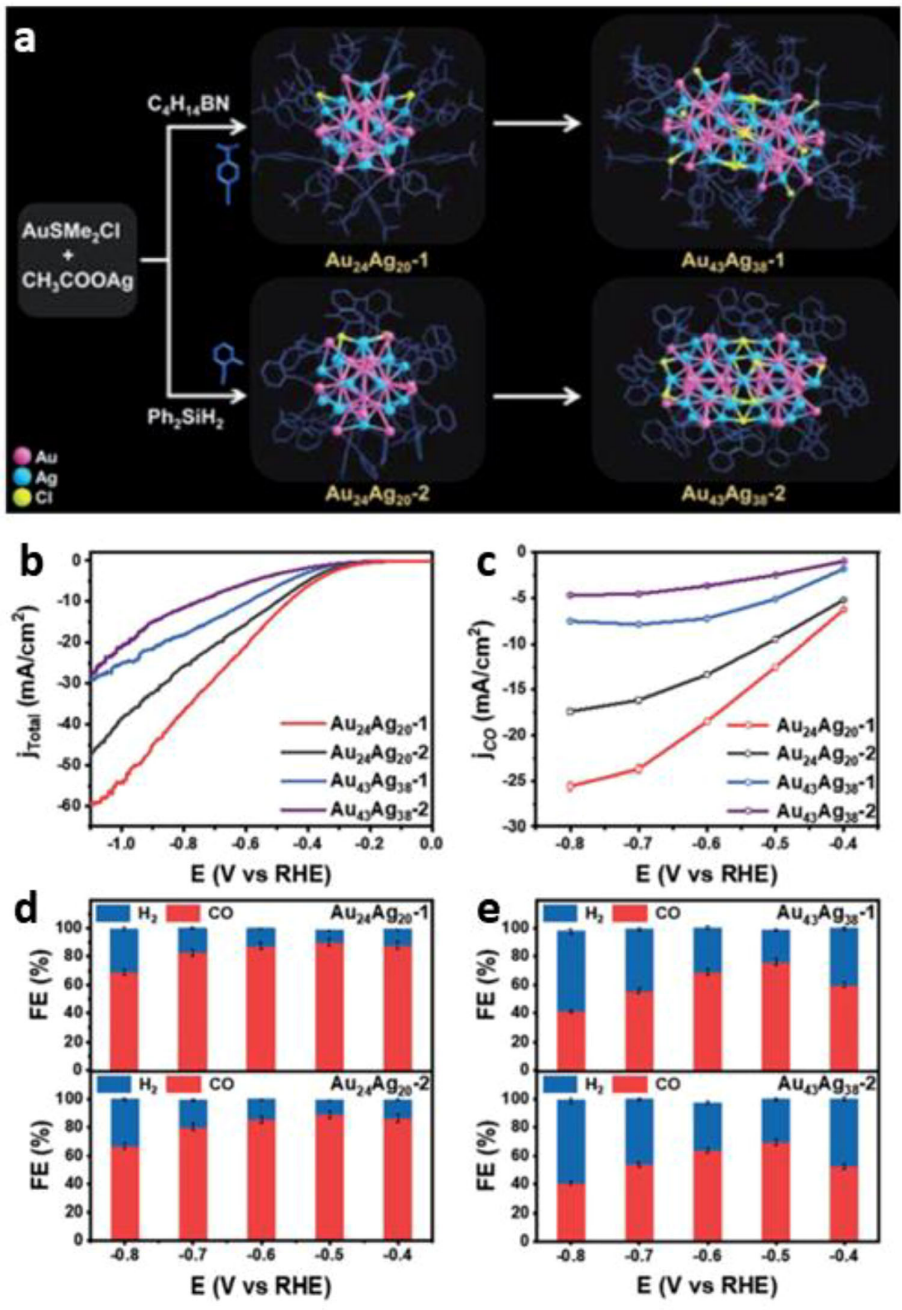
(a) Evolutionary scheme from monomeric Au_24_Ag_20_ NCs to dimeric Au_43_Ag_38_ NCs. evolved from the monomers. (b) LSV curves of the Au_24_Ag_20_ and Au_43_Ag_38_ NCs and (c) corresponding *j*_CO_. FE_CO_ and FE_H2_ on (d) Au_24_Ag_20,_ and (e) Au_43_Ag_38_ [81]. Copyright 2022, The Author(s).

Alloy NCs evolving from monomeric to dimeric structures have provided more details about the CO_2_RR mechanism. Two monomeric NCs, that is, Au_24_Ag_20_(C≡CPh‐*p*‐*^t^* Bu)_24_Cl_2_ (Au_24_Ag_20_‐1) and Au_24_Ag_20_(C≡CPh‐*o*‐CH_3_)_24_Cl_2_ (Au_24_Ag_20_‐2), can fuse via different pathways into dimeric Au_43_Ag_38_(CPh‐*p*‐*^t^* Bu)_36_Cl_12_ (Au_43_Ag_38_‐1) and Au_43_Ag_38_(C≡CPh‐*o*‐CH_3_)_36_Cl_9_ (Au_43_Ag_38_‐2), respectively (Figure 9a) [81]. Electrochemical testing revealed that within the applied potential window, monomers exhibited better FE_CO_ and *j*_CO_ compared to their dimeric counterparts. Specifically, at −0.5 V, monomeric NCs achieved FE_CO_ of 90%, whereas dimeric NCs showed merely 65% and 77% FE_CO_ for Au_43_Ag_38_‐1 and Au_43_Ag_38_‐2, respectively (Figure 9b–e) [81].

Moving on to Cu NCs, researchers synthesized a Cu_26_(dppe)_3_(CF_3_CO_2_)_8_(CH_3_O)_2_(C≡C^t^Bu)_4_H_11_^+^ (Cu_26_) NC co‐coordinated by phosphine, alkynyl, carboxylate, and methoxy ligands as well as hydrogen atoms through a gradient reduction strategy [83]. Cu_26_ possesses a Cu_4_@Cu_6_@Cu_16_ core‐shell‐shell structure, and the alkynyl ligands coordinate on the surface in two different ways. Phosphine‐stabilized Cu_25_H_22_(P(Ph‐*p*‐F)_3_)_12_ (Cu_25_), thiolate‐protected [Cu_61_(S*^t^* Bu)_26_S_6_Cl_6_H_14_]^+^ (Cu_61_), and alkynyl‐functionalized [Cu_53_(C≡C*^t^* Bu)_20_(CF_3_COO)_10_Cl_2_H_18_]^+^ (Cu_53_) were chosen to make the comparison. At −0.8 V, Cu_26_ achieved a FE_CO_ of 81% with minimal current decay during 50‐h electrolysis, whereas Cu_53_ exhibited much lower FE_CO_ [83].

**FIGURE 10:**
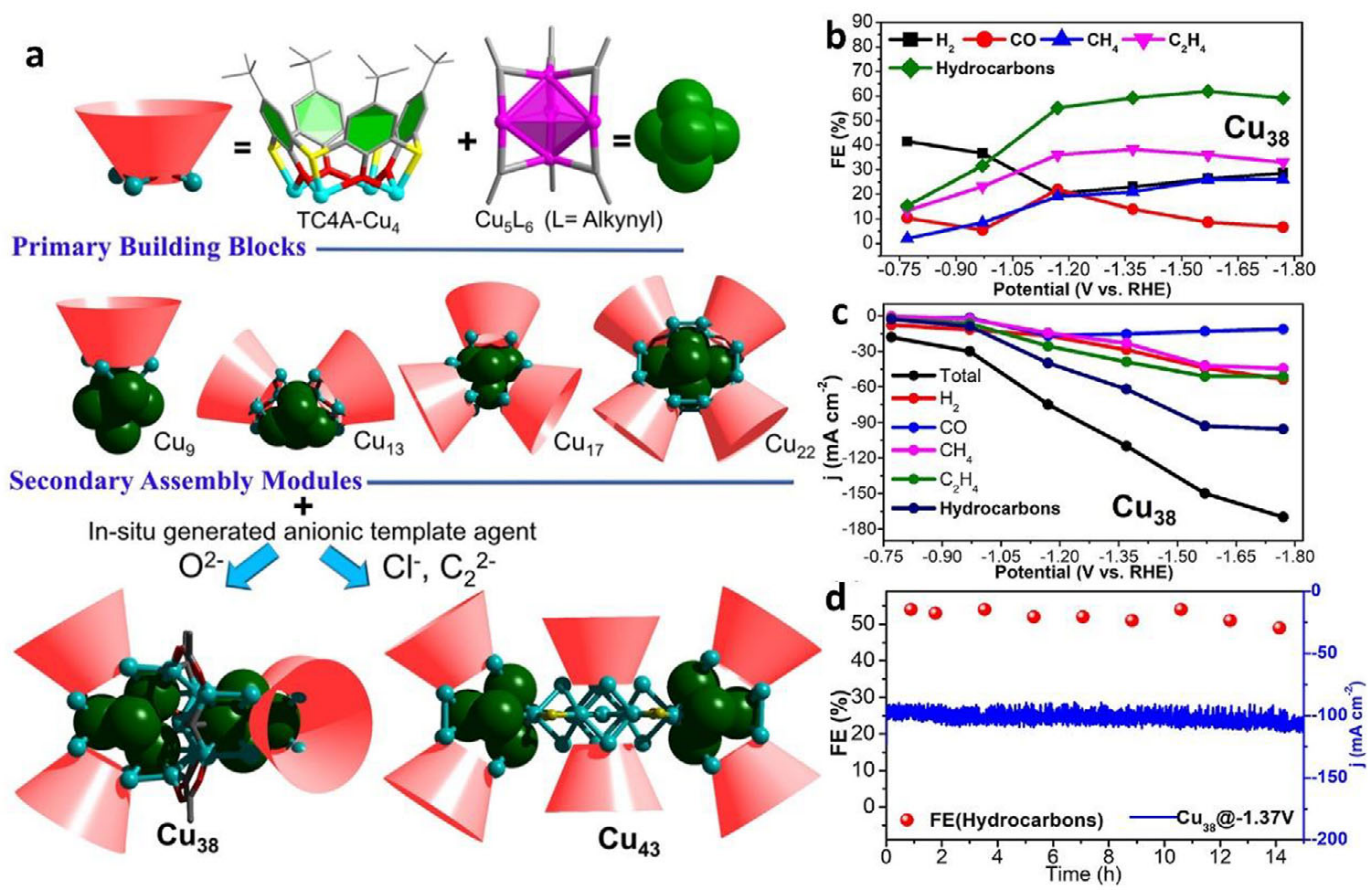
(a) Hierarchical Assembly Path of the TC4A/Alkynyl co‐protected Cu(I) NCs. (b) FEs and (c) current density of production at different working potentials for Cu_38_, and (d) stability test of Cu_38_ at −1.37 V for 15 h [84]. Copyright 2024, American Chemical Society.

High‐nuclearity Cu(I) NCs co‐protected by calixarene and alkynyl ligands, that is, Cu_38_O(TC4A)_4_(C≡CC_5_H_11_)_16_(OAc)_4_ (Cu_38_, TC4A = thiacalix[4]arene) were synthesized through a strategy of multiligand cooperative stabilization coupled with solvothermal in situ capture of anionic templates (Figure 10a). Subsequently, Cu_38_ and its two subunit NCs—NaCu_13_(TC4A)_2_(C≡C*^t^* Bu)_6_(CH_3_CN) and NaCu_17_(TC4A)_3_(C≡CC_5_H_11_)_6_—were employed as model catalysts for CO_2_RR [84]. Cu_38_ was found to efficiently reduce CO_2_ to hydrocarbons (FE_Hydrocarbon_ = 62%) with remarkable electrochemical stability (Figure 10b,c). After 15‐h electrolysis at −1.37 V, the Faradaic efficiencies for methane and ethylene exhibited only minor decay (55%–50%, Figure 10d). The results presented a marked contrast to previous reports on Cu‐based NCs, demonstrating their significant potential for electrochemical production of high‐value chemicals [84].

**FIGURE 11:**
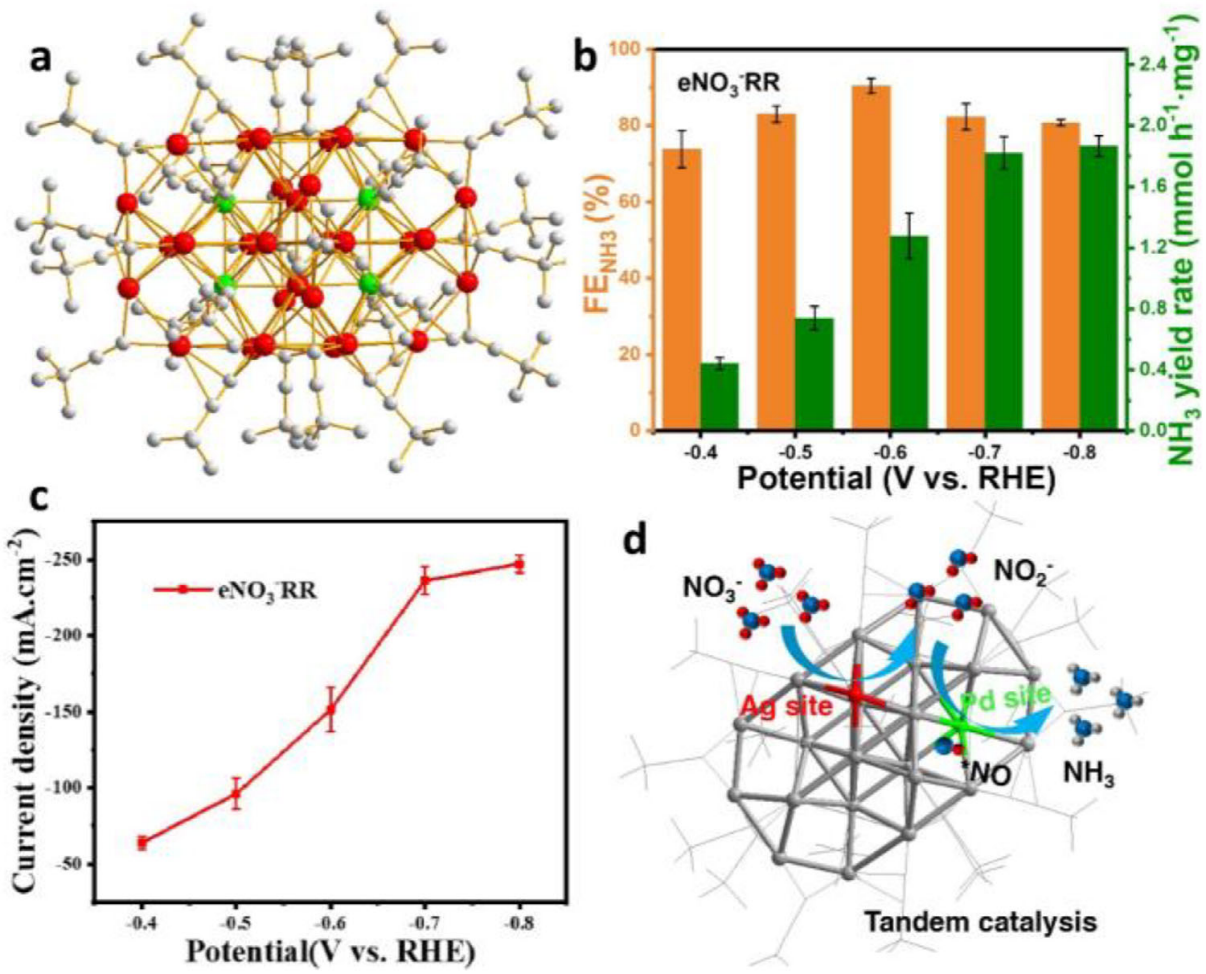
(a) Crystal structure of Ag_30_Pd_4_. (b) Potential‐dependent yield rate FE_NH3_ and (c) current density over Ag_30_Pd_4_ in NO_3_^−^RR. (d) Schematic diagram of the tandem catalytic mechanism of Ag_30_Pd_4_ catalyst [85]. Copyright 2023, American Chemical Society.

Recently, the electrochemical nitrate reduction reaction (NO_3_^−^RR) has garnered significant attention due to its dual capability to produce value‐added ammonia under mild conditions while simultaneously eliminating aqueous nitrate pollutants. Therefore, developing efficient and stable electrocatalysts for NO_3_^−^RR is imperative to provide a sustainable strategy for NH_3_ production. Atomically precise metal nanoclusters have demonstrated great potential for catalyzing NO_3_^−^RR. The first homoleptic alkynyl‐protected Ag–Pd NC—[Ag_30_Pd_4_(C≡C*^t^* Bu)_26_]^2+^ (Ag_30_Pd_4_) was synthesized and applied to NO_3_^−^RR (Figure 11a) [85]. Ag_30_Pd_4_ exhibited exceptional selectivity for nitrate reduction. In an H‐cell reactor with 1 M NaOH as the electrolyte, the FE_NH3_ reached 90% at −0.6 V (Figure 11b), accompanied by an average current density of 151 mA cm^−2^ (Figure 11c) and robust electrochemical stability. Significantly, in situ characterization further revealed that the Ag site is responsible for converting NO_3_^−^ into NO_2_^−^ and the Pd site plays the major role in catalyzing NO_2_^−^ into NH_3_. Thus, the whole reaction follows a tandem catalytic mechanism (Figure 11d) [85].

Subsequently, two alkynyl‐protected M_27_ NCs—Ag_18_Pd_9_(C≡CPhF)_24_ (Ag_18_Pd_9_) and [Ag_22_Pd_5_(C≡CPh(OMe)_2_)_26_]^−^ (Ag_22_Pd_5_)—were synthesized via ligand engineering [86]. When evaluated for NO_3_^−^RR, Ag_18_Pd_9_, despite its higher Pd content, exhibited significantly lower activity than that of Ag_22_Pd_5_. At −0.6 V, Ag_22_Pd_5_ achieved a FE_NH3_ of 94.4% and an ammonia production rate of 1.41 mmol·h^−1^·mg^−1^, which was over 3 times higher than that of Ag_18_Pd_9_ under identical conditions. The stark performance disparity was attributed to differences in the geometric configuration of the metal cores, emphasizing that structural modulation of NCs plays a more critical role in catalytic optimization than mere compositional variation [86].

Cu‐based NCs also demonstrated significant potential for NO_3_^−^RR. Homoleptic Ag_20_Cu_12_(C≡CAr^F^)_24_ (Ag_20_Cu_12_) exhibited excellent NH_3_ selectivity and yield in the electrocatalytic NO_3_^−^RR, achieving a high FE of 84.6% and a production rate of 0.138 mmol·h^−1^·mg^−1^ at –0.6 V, along with good electrochemical cycling stability [87]. The ammonia FE remained close to 80% even after five consecutive cycles. DFT calculations revealed the crucial role of Cu sites in facilitating NO_3_^−^ adsorption and enhancing NH_3_ selectivity [87].

Recently, the bulky 9‐HC≡C‐closo‐1,2‐C_2_B_10_H_11_ was used as ligands to prepare monomeric [Cu_13_(C_4_B_10_H_11_)_10_(PPh_3_)_2_(CH_3_CN)_2_]^3+^ (Cu_13_) and bridged dimeric [Cu_26_(C_4_B_10_H_11_)_16_(C_4_B_9_H_10_)_2_(PPh_3_)_2_(CH_3_CN)_4_]^4+^ (Cu_26_) NCs. Both Cu_13_ and Cu_26_ exhibited activity and selectivity for nitrate reduction to ammonia in an H‐type cell containing 0.1 M KNO_3_ in 0.5 M K_2_SO_4_ medium [88]. The FE_NH3_ of both NCs followed a volcano‐shaped trend with potential. Notably, Cu_26_ achieved a maximum FE_NH3_ of 85.1% at −0.85 V, significantly higher than that of Cu_13_ (50%). At all tested potentials, FE and NH_3_ production rate of Cu_26_ consistently surpassed those of Cu_13_, attaining a maximum NH_3_ production rate of 255 µmol·h^−1^·cm^−2^ [88]. Mechanistic insights from in situ infrared spectroscopy and theoretical calculations revealed that while the reaction pathways for Cu_13_ and Cu_26_ clusters are fundamentally similar, Cu_26_ exhibited enhanced kinetics in intermediate activation and ammonia formation during NO_3_^−^‐to‐NH_3_ conversion [88].

Finally, we cover some cases of using alkynyl‐protected NCs for the HER, which also holds significant importance in electrocatalysis. Although the HER mechanism has been extensively studied, unresolved challenges persist due primarily to atomic‐level heterogeneity in conventional catalysts. Au_25_(C≡CAr^F^)_18_ exhibited a 70 mV reduction in HER overpotential compared to Au_25_(SR)_18_, indicating enhanced catalytic activity of the Au_13_ core through ligand engineering [89]. By contrast, central Pt‐doped PtAu_24_(C≡CAr^F^)_18_ and PtAu_24_(SR)_18_ showed comparable HER activities, suggesting limited ligand effects on the Pt@Au_12_ core configuration [89].

Co‐coupling Mo_2_O_4_ structural units with Cu(I) alkynyl clusters can also improve the HER activity. One example is the Mo_2_O_4_Cu_17_(TC4A)_4_(PhC≡C)_6_ (Mo_2_Cu_17_) [90]. Experimental results demonstrated that Mo_2_Cu_17_ significantly enhanced HER performance compared to NaCu_13_(TC4A)_2_(PhC≡C)_4_(S*^t^* Bu)_2_(H_2_O) (Cu_13_) and Na_2_Cu_22_(TC4A)_4_(PhC≡C)_8_ (Cu_22_) NCs. In alkaline media, Mo_2_Cu_17_ achieved a current density of 10 mA cm^−2^ at an overpotential as low as 120 mV, outperforming Cu_13_ (178 mV) and Cu_22_ (214 mV). DFT calculations suggested that the higher activity of Mo_2_Cu_17_ arises from the synergistic coupling effect between the Mo_2_O_4_ subunit and Cu(I) sites [90].

Table 3 lists the published works focusing on using alkynyl‐protected NCs in electrochemical reduction reactions.

**TABLE 3:** Electrochemical CO_2_ reduction reactions catalyzed by alkynyl‐protected nanoclusters.

Catalyst	Reaction	Reactor	Electrolyte	Product	Activity FE (vs. RHE)	Partial current density (mA cm^−2^)	Product formation rate/TON	Stability	Refs.
Ag_32_(C≡CAr^F^)_24_	CO_2_RR	H‐Cell	0.5 M NaHCO_3_	CO	96.4% @ −0.8V	9.05 @ −1.0 V	NA	15 h @ −0.7 V	[69]
[Ag_15_(C≡C*^t^* Bu)_12_]^+^	CO_2_RR	H‐Cell	0.5 M KHCO_3_	CO	95% @ −0.6V	13 @ −0.9 V	6.47 mol_CO_ mol^−1^_NC_ s^−1^ @ –1.1V	10 h @ −0.75 V	[70]
[Au_7_Ag_8_(C≡C*^t^* Bu)_12_]^+^	CO_2_RR	GDE	1.0 M KOH	CO	98.1% @ −0.49V	155 @ −0.49 V	NA	10 h @ −0.49 V	[71]
[Ag_9_Cu_6_(C≡C*^t^* Bu)_12_]^+^				CO/HCO_2_H	94.2% @ −0.49 V (CO) 47% @ –1.19 V (HCO_2_H)	49.1 @ −1.19 V (HCO_2_H)	10 h @ −1.19 V	
[Au_2_Cu_5_Ag_8_(C≡C*^t^* Bu)_12_]^+^				CO/HCO_2_H	95% @ −0.49 V (CO) 28.3% @ –0.99 V (HCO_2_H)	32.7 @ −0.99 V (HCO_2_H)	10 h @ −0.99 V	
[ClAg_14_(C≡C*^t^* Bu)_12_]^+^	CO_2_RR	MEA	1.0 M KOH	CO	51% @ −2.6 V	400 @ −2.6 V	1400 A g^−1^	30 h @ 200 mA cm^−2^	[72]
		GDE			>95% @ −0.2 ∼ –0.6 V	285 @ –0.44 V	NA	NA	
		H‐Cell	0.05 M KHCO_3_ + 1.0 M KCl		87% @ −0.75 V	16 @ −0.75 V	8 mol_CO_ mol^−1^_NC_ s^−1^ @ –0.75V		
[Ag_9_Cu_6_(C≡CFc)_12_]^+^	CO_2_RR	MEA	0.1 M KHCO_3_	CO	99.4% @ −4.00V	680 @ −4.25 V	NA	200 h @ −3.0V	[73]
		H‐Cell			92% @ −0.77V	30 @ −1.04 V		NA	
[Ag_15_Cu_6_(C≡CR)_18_ (dppe)_2_]^−^	CO_2_RR	H‐Cell	0.1 M KHCO_3_	CO	91.3% @ −0.81 V	9 @ −0.81 V	NA	NA	[74]
		MEA			91.3% @ −3.5 V	154 @ −4.00 V	145 h @ 60 mA cm^−2^	
Ag_14_Cu_2_(C≡CR)_14_ (PPh_3_)_4_	CO_2_RR	GDE	1.0 M KOH	CO	83.7% @ −1.175V	68 @ −1.175 V	NA	10 h @ –0.975 V	[75]
Ag_19_Cu_2_(C≡CR)_12_ (PPh_3_)_6_Cl_6_	CO_2_RR	GDE	1.0 M KOH	CO	95.3% @ −1.37V	257 @ −1.3 V	NA	14 h @ −1.37V	[76]
[Au_15_Cu_4_(C≡CR) (dppm)_6_Cl_4_]^2+^	CO_2_RR	H‐Cell	0.1 M KHCO_3_	CO	89% @ −0.57V	5 @ −0.57 V	NA	NA	[77]
		MEA			90%–99% @ −2.75 ∼ –3.75V	413 @ −3.75 V		15 h @ 55 mA cm^−2^	
[Au_18_(dppm)_6_Br_4_]^2+^		H‐Cell			64% @ −0.81 V	4.5 @ −0.6 V		NA	
		MEA			60%–90% @ −2.75 V ∼ –3.75V	220 @ −3.75 V			
Au_44_(C_10_H_9_)_28_	CO_2_RR	H‐Cell	0.5 M KHCO_3_	CO	76%@ −0.6V	∼6 @ −0.8 V	NA	10 h @ −0.6 V	[78]
Ag_4+x_Au_40‐x_(C_10_H_9_)_28_					98% @ −0.5V	∼18 @ −0.8 V			
Au_43_(C≡C*^t^* Bu)_20_	CO_2_RR	H‐Cell	0.5 M KHCO_3_	CO	92.1% @ −0.57V	2.5 @ −0.57V	4718 h^−1^ @ −0.57V	2.5 h @ −0.57V	[79]
Au_42_Ag(C≡C*^t^* Bu)_20_					90.9% @ −0.57V	2.4 @ −0.57V	4458 h^−1^ @ −0.57V		
Au_24_Ag_20_(C≡CPh*^t^* Bu)_24_Cl_2_					72.9% @ −0.57V	1.3 @ −0.57V	2597 h^−1^ @ −0.57V		
Au_n_Ag_46−n_(C≡CR)_24_ (PPh_3_)_2_Cl_4_					59.5% @ −0.57V	0.8 @ −0.57V	1427 h^−1^ @ −0.57V		
Au_44_(C≡CR)_28_					74.5% @ −0.57V	1.0 @ −0.57V	2045 h^−1^ @ −0.57V	NA	
Au_36_(C≡CR)_24_					69.7% @ −0.57V	0.7 @ −0.57V	1325 h^−1^ @ −0.57V		
Au_23_(C≡CR)_15_					66.7% @ −0.57V	0.7 @ −0.57V	794 h^−1^ @ −0.57V		
Au_28_(C_2_B_10_H_11_S)_12_(tht)_4_Cl_4_	CO_2_RR	H‐Cell	0.5 M KHCO_3_	CO	98.5% @ −0.9 V	∼7.6 @ −0.9 V	NA	5.5 h @ −0.9 V	[82]
[Au_28_(C_4_B_10_H_11_)_12_(tht)_8_]^3+^					∼75% @ −0.8 V	∼3.8 @ −0.9 V	NA		
Au_24_Ag_20_(C≡CPh‐*p*‐*^t^* Bu)_24_Cl_2_	CO_2_RR	H‐Cell	0.5 M KHCO_3_	CO	90% @ −0.5V	25.5 @ −0.8 V	NA	NA	[81]
Au_43_Ag_38_(C≡CPh‐*p*‐*^t^* Bu)_36_Cl_12_					∼77% @ −0.5V	7.5 @ −0.8 V			
Au_24_Ag_20_(C≡CPh‐*o*‐CH_3_)_24_Cl_2_					90% @ −–0.5V	17.5 @ −0.8 V			
Au_43_Ag_38_(C≡CPh‐*o*‐CH_3_)_36_Cl_9_					∼65% @ −0.5V	5 @ −0.8 V			
[Cu_26_(dppe)_3_(TFA)_8_ (CH_3_O)_2_(TBA)_4_H_11_]^+^	CO_2_RR	H‐Cell	0.1 M KHCO_3_ +0.4 M KCl	CO	81% @ −0.8 V	2.5 @ −0.8 V	NA	50 h @ –0.8 V	[83]
Cu_38_O(TC4A)_4_ (C_5_H_11_C≡C)_16_(OAc)_4_	CO_2_RR	GDE	1.0 M KOH	CH_4_ C_2_H_4_	62.0% @ −1.57 V	94.9 @ −1.57 V (CH_4_+C_2_H_4_)	NA	15 h @ −1.37 V	[84]
NaCu_13_(TC4A)_2_ (C≡C*^t^* Bu)_6_(CH_3_CN)					14.4% @ −1.37 V	NA		NA	
NaCu_17_(TC4A)_3_(C≡CC_5_H_11_)_6_					27.9% @ −1.57 V				
[Ag_30_Pd_4_(C≡C*^t^* Bu)_26_]^2+^	NO_3_^–^RR	H‐Cell	1.0 M NaOH	NH_3_	90.3% @ −0.6 V	151 @ −0.6 V	1.28 mmol·h^−1^·mg^−1^ @ –0.6 V	5 cycles @ –0.6V	[85]
[Ag_22_Pd_5_(C≡CPh(OMe)_2_)_26_]^–^	NO_3_^–^RR	H‐Cell	1.0 M NaOH	NH_3_	94.4% @ −0.6 V	155 @ −0.6V	1.41 mmol·h^−1^·mg^−1^ @ –0.6 V	5 cycles @ –0.6V	[86]
Ag_18_Pd_9_(C≡CPhF)_24_					43.9%@ −0.5 V	40 @ −0.6V	0.41 mmol·h^−1^·mg^−1^ @ –0.5 V	NA	
Ag_20_Cu_12_(C≡CAr^F^)_24_	NO_3_^–^RR	H‐Cell	0.5 M Na_2_SO_4_	NH_3_	84.6% @ –0.6 V	NA	138 µmol·h^−1^·mg^−1^ @ –0.6 V	5 cycles	[87]
[Cu_13_(C_4_B_10_H_11_)_10_(PPh_3_)_2_ACN_2_]^3+^	NO_3_^–^RR	H‐Cell	0.5 M K_2_SO_4_	NH_3_	50% @ −0.85 V	∼23 @ −0.85 V	145 µmol h^−1^ cm^−2^ @ –1.05 V	5 cycles @ −0.85 V	[88]
[Cu_26_(C_4_B_10_H_11_)_16_(C_4_B_9_H_10_)_2_ (PPh_3_)_2_ACN_4_] [4]					85.1% @ −0.85 V	∼28 @ −0.85 V	255 µmol h^−1^ cm^−2^ @ –1.05 V		
Au_25_(C≡CAr^F^)_18_	HER	Cell	0.5 M H_2_SO_4_	H_2_	NA	10 @ −0.5 V	289 mol_H2_/(mol_cat_·s)	NA	[89]
PtAu_24_(C≡CAr^F^)_18_						10 @ −0.44 V	NA		
[Mo_2_O_4_Cu_17_(TC4A)_4_ (C≡CPh)_6_]	HER	Cell	1.0 M KOH	H_2_	NA	10 @ −0.120 V	131.8 mV dec^−1^ (Tafel slope)	12 h @ −0.12 V	[90]
NaCu_13_(TC4A)_2_(C≡CPh)_4_ (S*^t^* Bu)_2_(H_2_O)						10 @ −0.178 V	196.3 mV dec^−1^	NA	
Na_2_Cu_22_(TC4A)_4_ (C≡CPh)_8_						10 @ −0.214 V	258.2 mV dec^−1^		

## 5 Conclusion and Perspective

Since the first time that atomically precise coinage metal NCs were used as model systems in electrocatalysis [87], these catalysts have been designed to be versatile with tunable activities over the past decade, fully utilizing their advantages of atomic precision—atomically resolved structures, discrete electronic states, and tailorable surfaces, providing unprecedented opportunities to establish correlations between the atomic structure and catalytic behavior—an achievement difficult to realize by conventional nanoparticle systems.

In this article, we have overviewed the effects of size, ligand type, morphology, core doping, and surface modification that influence electrocatalytic activity and selectivity, especially for CO_2_RR, and then highlighted cases showing superior performance catalyzed by non–alkynyl‐protected NCs. The Cu‐based NCs for CO_2_RR were emphasized because they are promising candidates to produce value‐added multi‐carbons.

The alkynyl‐protected NCs have opened a new dimension with unique σ–π anchoring of alkynyl ligands, which reduces surface coverage and exposes more active sites, thereby enhancing catalytic activity. The ability to integrate functional ligands and to construct bimetallic or unique architectures has significantly improved the electrocatalytic properties of NCs not only for CO_2_RR, but also NO_3_^−^RR and HER, demonstrating the central role of surface chemistry in dictating catalytic outcomes.

Several directions merit further exploration in future efforts:

1. Expanding ligand chemistry beyond homoleptic stabilization. Combining alkynyl ligands with π‐conjugated systems or multidentate organic linkers may enable greater exposure of metal atoms as surface active sites and facilitate capture and activation of reactants.
2. Bridging the gap between model systems and practical electrocatalysts. Integrating atomically precise NCs into scalable electrodes and applying them to industrial standards will be crucial for translating fundamental research into real‐world electrocatalytic devices.
3. Cu‐based nanocluster electrocatalysts need to be explored more due to copper's intrinsic capability of C–C coupling to convert CO_2_ to multicarbon products of higher value. Unlike the consistent observations in Au NCs that lattice hydrides are beneficial for higher FE_CO_, the presence of lattice hydrides on the Cu surface does not always lead to C_2_ generation. Designing more stable Cu‐based NCs with improved ligand‐shell engineering would benefit the production of more valuable CO_2_RR products.
4. Rational design of heterometallic NCs. Precise replacement of metal atoms with heteroatoms can enable bifunctional catalysis, as exemplified by Ag–Pd and Au–Cu systems. Extending this concept to more complex multimetallic combinations may unlock new reaction pathways.
5. Beyond the reactions such as oxygen evolution reaction, HER, CO_2_RR, and NO_3_^−^RR that have been performed on atomically precise NCs, other electrocatalysis, including nitrogen reduction reaction, organic electrosynthesis (C–C and C–X coupling reactions), can be further exploited to expand the catalytic applications of NC catalysts.

In conclusion, coinage metal NCs of atomic precision represent a versatile platform for electrocatalysis. By uniting precise structural control with innovative ligand chemistry and advanced characterization, the field is poised to move beyond proof‐of‐concept demonstrations and move toward the rational design of next‐generation, high‐efficiency, and sustainable electrocatalysts.

## Conflicts of Interest

The authors declare no conflicts interests.

## Data Availability

The data that support the findings of this study are available in the supplementary material of this article.
